# Generation and validation of a novel multitarget small molecule in glioblastoma

**DOI:** 10.1038/s41419-025-07569-1

**Published:** 2025-04-04

**Authors:** Aizpea Artetxe-Zurutuza, Nerea Iturrioz-Rodriguez, Joseba Elizazu, Mireia Toledano-Pinedo, Alicia Porro-Pérez, Irati De Goñi, Alejandro Elua-Pinin, Linda Schäker-Hübner, Mikel Azkargorta, Felix Elortza, Isabel Iriepa, Francisco Lòpez-Muñoz, Veronica Moncho-Amor, Finn K. Hansen, Nicolás Sampron, Jose Luis Marco-Contelles, Ander Matheu

**Affiliations:** 1 Cellular Oncology group, Biogipuzkoa (Biodonostia) Health Research Institute, San Sebastian, Spain; 2 Laboratory of Medicinal Chemistry (Institute of General Organic Chemistry, CSIC), Madrid, Spain; 3 Neurosurgery Service, Donostia University Hospital, San Sebastian, Spain; 4 Department of Pharmaceutical and Cell Biological Chemistry, Pharmaceutical Institute, University of Bonn, Bonn, Germany; 5 Proteomics Platform, CIC bioGUNE, Basque Research and Technology Alliance (BRTA), CIBERehd, Derio, Spain; 6 Alcala University, Department of Organic and Inorganic Chemistry, Andrés M. del Río Chemistry Research Institute (IQAR); and DISCOBAC group, Castilla-La Mancha Health Research Institute (IDISCAM), Madrid, Spain; 7 Faculty of Health Sciences–HM Hospitals, Camilo José Cela University; HM Hospitals Health Research Institute; Neuropsychopharmacology Unit, “Hospital 12 de Octubre” Research Institute, Madrid, Spain; 8 Centre for Biomedical Network Research on Rare Diseases (CIBERER), ISCIII, Madrid, Spain; 9 Centre for Biomedical Network Research on frailty and healthy aging (CIBERFES), ISCIII, Madrid, Spain; 10 IKERBASQUE, Basque Foundation for Science, Bilbao, Spain

**Keywords:** CNS cancer, Cancer stem cells

## Abstract

The development of multitarget small molecules (MSMs) has emerged as a powerful strategy for the treatment of multifactorial diseases such as cancer. Glioblastoma is the most prevalent and malignant primary brain tumor in adults, which is characterized by poor prognosis and a high heterogeneity. Current standards of treatment present limited effectiveness, as patients develop therapy resistance and recur. In this work, we synthesized and characterized a novel multi-target molecule (named DDI199 or contilistat), which is a polyfunctionalized indole derivative developed by juxtaposing selected pharmacophoric moieties of the parent compounds Contilisant and Vorinostat (SAHA) to act as multifunctional ligands that inhibit histone deacetylases (HDACs), monoamine oxidases (MAOs) and cholinesterases (ChEs), and modulate histamine H3 (H3R) and Sigma 1 Receptor (S1R) receptors. DDI199 exerts high cytotoxic activity in conventional glioblastoma cell lines and patient-derived glioma stem cells in vitro. Importantly, it significantly reduces tumor growth in vivo, both alone and in combination with temozolomide (TMZ). The comparison with SAHA showed higher target specificity and antitumor activity of the new molecule. Transcriptomic and proteomic analyses of patient-derived glioma stem cells revealed a deregulation in cell cycle, DNA remodeling and neurotransmission activity by the treatment with DDI199. In conclusion, our data reveal the efficacy of a novel MSM in glioblastoma pre-clinical setting.

## Introduction

The development of MSMs has emerged as a powerful tool for the identification of new drugs for the treatment of multifactorial diseases [1], including cancer [2] as well as neurodegenerative diseases such as Alzheimer’s (AD) or Parkinson’s diseases [3]. This is based on the shared pathological mechanisms between cancer and other diseases such as neurodegenerative [2].

Indole is recently gaining interest for the development of new compounds. Indole is a naturally occurring heterocyclic compound that presents good bioavailability and pharmacological activities. Thus, it has been proposed as a promising scaffold for the development of novel drugs for the treatment of different diseases. As a result, we generated the MSM Contilisant*,* an indole-based tetratarget compound, combining histamine H3 receptor (H_3_R)/sigma 1 receptor (S_1_R) modulation with AChE/BChE, and MAO A/B inhibition for AD [4]. More recently, in order to perform the pharmacological optimization of Contilisant, we have incorporated new pharmacophoric groups to produce novel pharmacological effects.

In this context, the inhibition of histone deacetylases (HDACs) offers a promising alternative that warrants further investigation for multifactorial diseases [5, 6]. To date, 11 HDACs have been identified. They are divided into four classes according to their phylogenetic sequence, localization and function; class I (HDACs 1, 2, 3, and 8), class IIa (HDACs 4, 5, 7, and 9), class IIb (HDACs 6 and 10), and class IV (HDAC11) [7]. Overexpression of HDACs has been associated with various types of cancers, such as solid and haematologic malignancies, including glioblastoma. Importantly, the efficacy of HDAC inhibitors (HDACis), in particular pan-HDACis such as SAHA, for the treatment of haematologic cancers has been well documented, being already the first molecule approved by the FDA for the treatment of advanced primary cutaneous T cell lymphoma [8]. With respect to its efficacy in glioblastoma, several pre-clinical and clinical trials revealed positive anti-tumor effects and postulated that it could be a promising agent in brain tumors [9, 10]. Regarding AD, SAHA restores contextual memory in APP/PS1 transgenic AD mice [11].

Glioblastoma is the most common and malignant primary brain tumor with prognosis of the patients remaining very poor [12]. Current standard of treatment for GBM is based on the surgical resection of the tumor, followed by radiotherapy and chemotherapy with TMZ. However, the high intra- and inter-tumoral heterogeneity, the presence of glioma stem cells and the heterogeneous tumor microenvironment leads to therapy resistance and recurrence [13, 14]. Thus, the development of novel effective treatments is still needed. For that purpose, and given the high heterogeneity of glioblastoma, the development of multi-target-directed therapies have emerged as a promising approach [15]. Indeed, several indole-based compounds have been synthesized, which are presenting promising results both in pre-clinical and clinical trials [16].

Taking into account the challenge of high heterogeneity in GBM, the future of indole as a scaffold for drug development, the promising results observed with Contilisant in AD and the impact of HDACs in multiple diseases, including cancer and neurodegenerative diseases, we generated a novel compound named DDI199 (also named contilistat) by adding a structure mimicking SAHA to Contilisant [17]. This multitarget approach and design has gained strong support from recent studies. Thus, Yao and colleagues reported HDAC1/MAO-B dual inhibitors against AD [18]. In relation to brain tumors, other group designed hydroxamate-based hybrids using AChEis like donepezil or memantine to modulate S1R and HDAC isoforms exerting anti-glioblastoma effects [19, 20]. Additionally, *N*-Methylpropargylamine-conjugated hydroxamic acids have been generated as dual inhibitors of MAO-A and HDAC for glioma treatment [21]. For other cancers, a MAOi pargyline into an HDAC inhibitory template have been installed that resulted in the identification of a promising antiprostate cancer agent [22].

In the present study, we report the design and synthesis of a novel indole-based multi-target directed molecule, we characterize the expression of its targets in different human GBM cohorts, and test its effect in glioblastoma both by in vitro and in vivo experiments.

## Materials And Methods

### Human cohorts and survival analysis

RNAseq data by the TCGA and GTEx were obtained from (https://xenabrowser.net/). This data includes a total of 165 glioblastoma samples with 207 normal tissue samples form brain cortex, 414 tumor and 36 normal tissue samples for stomach adenocarcinoma (STAD), 515 tumor and 59 normal tissue samples for lung adenocarcinoma (LUAD) and 179 tumor and 171 normal samples for pancreatic adenocarcinoma (PAAD). CGGA RNAseq, including 225 glioblastoma and 20 non-glioma samples was downloaded from (https://www.cgga.org.cn/). For the Rembrandt, Joo and Gill cohorts, microarray and RNAseq (Gill) gene expression data were downloaded from Gliovis (http://gliovis.bioinfo.cnio.es/). The Rembrandt cohort includes 219 glioblastoma and 28 non-tumor samples, the Joo includes 57 glioblastoma, and Gill 75 glioblastoma and 17 non-tumor samples. Normal versus tumor expression was tested using the Welch t-test. For survival analyses, the best cutoff method by the R package *survminer* was used to categorize the samples by high and low expression. Kaplan-Meier curves were created using the same package, and statistical analyses were done with cox regression.

The Human Protein Atlas brain scRNAseq dataset was obtained from https://www.proteinatlas.org/about/download. The analysis includes a total of 76,533 cells from the primary motor cortex cells of 2 human brains. The scRNAseq from glioblastoma was obtained from (https://cellxgene.cziscience.com/), which comprises 338,542 glioblastoma cells from 110 donors/patients across 16 independent studies. Data was loaded into R and the “cell_type” annotation was used for analysis.

### Cell lines and cultures

Patient-derived glioma stem cell lines (GNS166 and GNS179) were kindly provided by Dr. Steve Pollard and cultured in adhesion in Laminin with DMEM/F12 media (Gibco) supplemented with 100 U/mL penicillin and streptomycin, 2 mM of L-Glutamine, 1% N2 (Gibco), 2% B27 (Gibco), 1.34% D-(+)-Glucose solution 45% (Sigma-Aldrich), 20 ng/mL basic Fibroblast Growth Factor (bFGF, Gibco) and 20 ng/mL epidermal growth factor (EGF, Sigma-Aldrich).

U87-MG and U251-MG glioma cells were purchased from ATCC, while pancreatic cell line RWP1 was kindly provided by Dr. Francisco Real. These lines were cultured in DMEM (Gibco) supplemented with 10% FBS, 100 U/mL penicillin and streptomycin and 2 mM of L-Glutamine. Normal Human Astrocytes (NHA) were purchased from ScienCell and cultured with an astrocyte medium kit (ScienCell) supplemented with FBS, penicillin/streptomycin and Astrocyte Growth Supplement (AGS). Finally, lung cancer NCI-H1299 cells purchased from ATCC and gastric cancer MKN45 cells obtained from the Leibniz Institute DSMZ-German Collection of Microorganisms and Cell Cultured were cultured in RPMI (Gibco) supplemented with 10% FBS, 100 U/mL penicillin and streptomycin and 2 mM of L-Glutamine. All cell lines were maintained at standard conditions of 37 °C and 5% CO_2_ in humidified atmosphere, and regular tests were done to verify they were Mycoplasma free.

For oncosphere formation assay, cells were cultured in non-treated 48-well plates in DMEM/F12 medium supplemented with EGF and FGF and fresh media was added every 2 days. After 7 days, oncosphere formation was documented and the number of spheres was quantified using Qupath.

### Compounds

DDI199 was prepared as described [17]. SAHA (Cayman) and Tubastatin A (Cayman) were used as HDAC pan-inhibitor and specific HDAC6 inhibitor, respectively. For the case of MAO inhibition studies, Clorgiline (Sigma-Aldrich) was used as MAO-A inhibitor, and Selegiline (Sigma-Aldrich) and Rasalgiline (Sigma-Aldrich) for MAO-B. Last, compounds for AChE were Donepezil (Sigma-Aldrich) and Rivastigmine (Sigma-Aldrich), the second one been also used for BChE. Additionally, TMZ (Sigma-Aldrich) was used. All compounds were dissolved in DMSO, with the exception of Donepezil and Rasalgiline, which were dissolved in distilled water, and Rivastigmine, which was dissolved in DPBS.

### Cell viability assay

MTT assay was used to study cell viability. For that, cells were seeded at a density of 1.5 × 10^3^ cells/well in 96-well plates, incubated overnight and then treated with increasing concentrations of DDI199. After 72 h of treatment, MTT absorbance was measured at 570 nm. GraphPad Prism software was used to analyze results and calculate IC_50_ values.

### Cell cycle analysis by flow cytometry

Cells were harvested using accutase and washed with PBS. 1·10^6^ cells were fixed with 70% cold ethanol at −20 °C for at least 24 h, and incubated with 0.5% Triton X-100 and 25 µg/mL RNase A in PBS for 30 min at room temperature. Then, DNA was stained with 25 ng/mL propidium iodide for 15 min and samples were analyzed using a CytoFLEX flow cytometer (Beckman Coulter Co.).

### EdU cell proliferation assay

*Click-iT™ EdU Cell Proliferation Kit for Imaging* (Invitrogen) was used according to the manufacturer’s instructions. Briefly, GNS166 cells were seeded and maintained overnight before DDI199 treatment. After 48 h, fresh culture medium containing 10 μM of EdU was added for 24 h and then, cells were fixed with 4% paraformaldehyde. Axio Observer 7 epifluorescence microscope was used to take pictures, which were later quantified in Qupath.

### Western blot

Protein lysates were resolved using sodium dodecyl sulfate-polyacrylamide gel electrophoresis. Primary antibodies against HDAC1 (Ab109411, abcam), HDAC6 (7558S, Cell Signaling), acetyl H3 (9649, Cell Signaling), acetyl α-tubulin (Ab24610, abcam), acetylated-Lysine (9441, Cell Signaling) and β-actin (A5441, Sigma-Aldrich) were used. Membranes were then incubated with the corresponding anti-rabbit (7074S, Cell Signaling) or anti-mouse (7076S, Cell Signaling) secondary antibody conjugated to horseradish peroxidase (HRP), and NOVEX ECL Chemi Substrate (ThermoFisher) or SuperSignal West Femto Maximum Sensitivity Substrate (ThermoFisher) was used for protein detection by iBright imaging system.

### Enzyme inhibition studies

The inhibitory capacity of DDI199 on HDACs was studied as previously described [23]. The capacity of DDI199 to inhibit MAOs was studied by mimicking the reaction occurring in cells. Briefly, inhibitor solution containing 10 µM of DDI199 or reference compound was incubated for 30 minutes at 37 °C together with the enzyme solution, containing either recombinant hMAO-A (Sigma-Aldrich) at 0.5 U/mL or hMAO-B (Sigma-Aldrich) at 1.5 U/mL. 200 U/mL horseradish peroxidase (Sigma-Aldrich), 20 mM Ampliflu Red (Sigma-Aldrich) and 100 mM tyramine hydrochloride (Sigma-Aldrich) were then added and absorbance at 570 nm was measured every 5 minutes for 30 minutes. Percentage of inhibition was estimated from these values. In the case where the inhibition percentage was higher than 50%, the IC_50_ was measured by repeating the same protocol using increasing concentrations of the inhibitor compound.

Similarly, Ellman’s colorimetric method was used to study the inhibitory capacity of the compound on ChEs. Enzyme solution containing either recombinant hAChE (Sigma-Aldrich) at 0.5 U/mL or hBChE (Sigma-Aldrich) at 0.25 U/mL was incubated with inhibitor solution containing 10 µM of DDI199 or reference compound and 0.3 mM of DTNB (Sigma-Aldrich) for 20 minutes at 37 °C. Substrate solution of acetylthiocholine iodide (Sigma-Aldrich) or butyrylthiocholine iodide (Sigma-Aldrich) at 1.5 mM was then added and absorbance at 405 nm was measured every minute for 10 minutes. After the estimation of the inhibition percentage, IC_50_ was measured in cases presenting more than 50% of inhibition. In addition, kinetic of enzyme inhibition was also studied by repeating the same protocol and using five different concentrations of the substrate and four different concentrations of the inhibitor. Results were represented in a Lineweaver-Burk plot using GraphPad Prism software, and the constant of inhibition (Ki) was estimated from the point at which the line cuts the x-axis at the secondary plot.

### Immunofluorescence

Immunofluorescence assays were performed following standard procedures. Cells were seeded at a density of 2 × 10^4^ cells/well in 8-well immunofluorescence chambers (LabTek Thermo) and incubated overnight before treating with increasing concentrations of DDI199. After 48 h, cells were fixed with 4% PFA. Primary antibodies against phosphohistone H3 (pH3) (ab14955, abcam), Cleaved Caspase 3 (AF385, R&D Systems), Ki67 (ab15580, abcam) and PARP1 (ab32064, abcam) were used. Secondary antibodies were Alexa Fluor anti-mouse (A21422, Invitrogen) and Alexa Fluor anti-rabbit (A31572, Invitrogen), and chromatin was stained with DAPI (D9541, Sigma-Aldrich). Axio Observer 7 epifluorescence microscope was used to take pictures, which were later quantified in Qupath.

### RNAseq and proteomic studies

GNS166 cells were cultured with 5 µM of DDI199 for 48 h, and pellets were then taken for RNAseq and proteomics. Total RNA was extracted using Trizol (Life Thechnologies) and samples were sent to BGI Tech Solutions for transcriptomic analysis. 44 million clean reads per sample were generated on average, and DESeq2 package (version 1.44.0) was used for differential expression analysis using default settings. Genes presenting a fold-change value ≥ 4 or ≤ −4 and a FDR-adjusted *P* value < 0.01 were defined as differentially expressed and used for downstream enrichment studies. The data discussed in this publication have been deposited in NCBI’s Gene Expression Omnibus and are accessible in GSE282561.

Proteomic studies were performed by the Proteomics Facility of the CIC bioGUNE by label-free relative quantification on nLC MS/MS. R package *clusterProfiler* was used for overrepresentation analysis of significantly differentially expressed proteins (*p* < 0.05), which was performed independently for upregulated and downregulated proteins. In both approaches, the top 20 enriched terms were plotted and the pathways were clustered using divisive hierarchical clustering based on pairwise Jaccard distances.

A combined study of both omic approaches was performed. For that, the enrichment analyses both on the proteome and the RNAseq studies was done on all three Gene Ontology domains, using all genes as background, and independently for upregulated and downregulated genes and proteins. Gene sets were then clustered using the Jaccard distances between the gene sets. In detail, the Jaccard similarity matrix of the top 25 enriched gene sets was loaded into the R package *igraph* (version 2.0.1.1) as a graph, and after deleting the edges with less than a 0.2 similarity index, the Louvain algorithm with a resolution parameter of 0.3 was used to construct the clusters. Then, the genes and proteins that were significantly expressed in both approaches were selected for each cluster and subjected to spearman correlation analyses with the target genes of DDI199 in TCGA cohort. The mass spectrometry proteomics data have been deposited to the ProteomeXchange Consortium via the PRIDE partner repository with the dataset identifier PXD058898.

### RNA extraction and q-RTPCR

Total RNA was extracted using Trizol (Life Thechnologies). Reverse transcription was then performed with the Maxima First Strand cDNA Synthesis Kit (ThermoFisher), following manufacturer’s guidelines. Quantitative real-time PCR was carried out in a CFX384 Thermal Cycler (BioRad) by Absolute SYBR Green mix (ThermoFisher). GAPDH was used as housekeeping gene for the correction of variations on initial cDNA levels, and relative gene expression was determined using the 2^-ΔΔCt^ formula.

### In vivo carcinogenesis assays

For tumor growth experiments, 5 × 10^5^ U87-MG cells were subcutaneously injected into two bottom flanks of athymic nude-Foxn1^nu^ mice, and once tumors reached a size of 25-50 mm^3^, mice were sorted into groups taking into account gender and tumor size. Afterwards, mice were intratumorally treated with vehicle, SAHA 100 µM or DDI199 100 µM following a schedule of 5 days on and 2 days off. In combined treatment, mice were treated intraperitoneally with 0.5 mg/Kg of TMZ for 5 days and intratumorally following a schedule of 5 days on and 2 days off with vehicle, SAHA 100 µM or DDI199 100 µM. After 4 weeks of treatment, some mice were kept for washout experiment, while the rest were sacrificed and tumors were extracted for immunohistochemistry and to disaggregate for reinjection and oncosphere formation studies. For reinjection assays, 1.5 × 10^5^ cells were subcutaneously injected and tumor size was periodically measured. Once tumors reached end-point criteria, they were extracted for immunohistochemical analysis. An external caliper was used for tumor size measurement and the tumor volume was estimated using the formula V = L · W^2^ · 0.5 (where L is tumor length and W is the tumor width).

### In vivo toxicity assay

15 mg/mL of DDI199 or vehicle (DMSO) were intraperitoneally injected in athymic nude-Foxn1^nu^ mice following a schedule of 5 days on and 2 days off and changes in body weight were measured to assess the potential toxicity of the MSM. At the end-point macroscopic analysis of the different organs was done.

### Immunohistochemistry

Subcutaneous tumors were extracted, fixed with 4% formaldehyde for 48 h and embedded in paraffin. Tumor sections were obtained and stainings for acetyl H3 (9649, Cell Signaling), acetylated α-tubulin (ab179484, abcam), PCNA (Ab18197, abcam) and Cleaved Caspase 3 (AF835, R&D Systems) were performed. ZEISS Axioscan 7 slide scanner was used to capture images of the stainings and analyses were done using Qupath.

### Online available tools for DDI199 characteristics

BBB permeability with BBB Predictor (https://www.cbligand.org/BBB/predictor.php) and physicochemical properties with Molinspiration Calculation of Molecular Properties toolkit (https://www.molinspiration.com/cgi/properties). DDI199 chemical structure was introduced and prediction was done maintaining the parameters established by default in the tools.

### Statistical analysis

Statistical analyses of the computational study of this work were performed using R version 4.4.1. and the statistical method specified on each section. Regarding to the experimental part, GraphPad Prism software (Version 8.3.0, San Diego, CA, USA) was used for statistical analyses. Results were represented as mean values ± standard error of the mean (SEM). To calculate statistical significance, ANOVA test was performed to compare differences between 3 or more groups and Student *t* test for 2. Each column was compared with the mean of the control column, and Dunnet test was used as method to correct multiple comparisons. In the case of in vivo tumor growth analysis, two-way ANOVA test using the mixed-effects model method was performed. Differences were considered statistically significant (#, *P* ≤ 0.1, *p* ≤ 0.05 *, *p* ≤ 0.01 ** and *p* ≤ 0.001 ***). It is indicated in the text in cases where additional tests have been performed.

## Results

### Expression of HDACs, MAOs and ChEs is altered in human glioblastoma samples

**Fig. 1:**
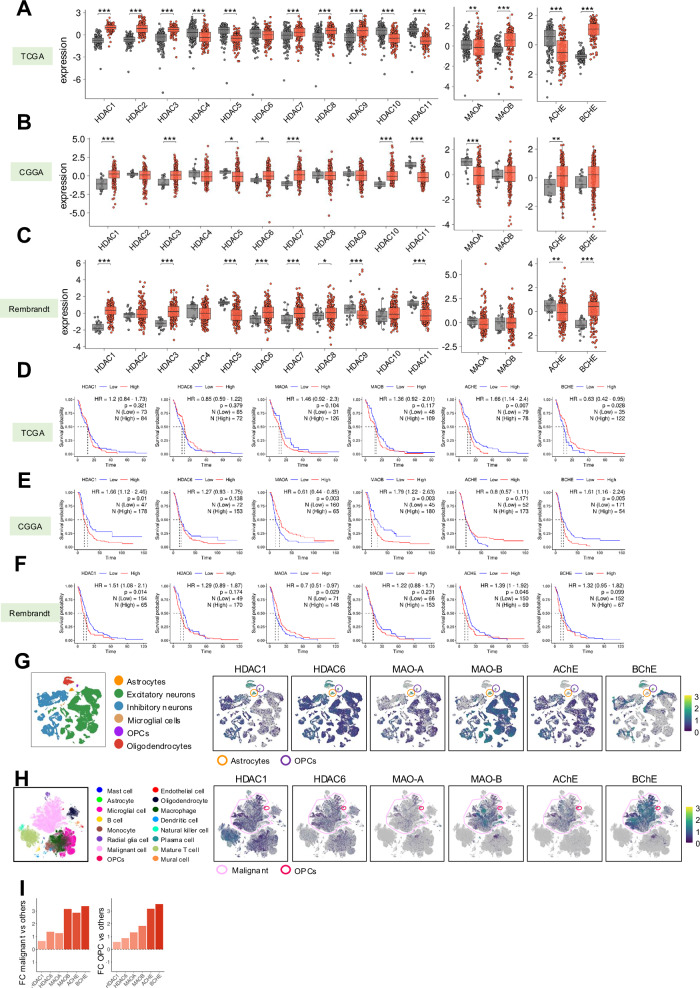
High levels of target genes in glioblastoma samples. **A-C** Expression of target genes in normal tissue (grey) versus glioblastoma samples (red) in TCGA, CGGA and Rembrandt cohorts. **D-F** Kaplan-Meier survival curves of patients with low vs high expression of target genes in TCGA, CGGA and Rembrandt cohorts. **G** Expression of target genes in cell types from single-cell RNAseq of healthy brain tissue from The Human Protein Atlas. **H** Expression of target genes in cell types from single-cell RNAseq of glioblastoma samples. **I** Mean log2-fold change of expressing cells in Malignant cells and OPCs versus other cell types.

To further characterize the expression level of the target genes, publicly available data of different large independent cohorts such as TCGA, CGGA and Rembrandt were used. First, the expression of HDACs was compared between glioblastoma samples and healthy brain tissue. The levels of the different HDACs were significantly altered in the different cohorts (Fig. 1A-C and Fig. Suppl. 1A), with several HDACs, mainly HDAC1, HDAC3, HDAC6 and HDAC7 being increased in tumor samples. With regard to MAOs and ChEs, both were deregulated in glioblastoma samples being MAO-B and BChE highly expressed in tumor samples in most cohorts (Fig. 1A-C and Fig. Suppl. 1A). Among the different HDACs, previous studies have also shown that HDAC1 and HDAC6 are overexpressed in glioblastoma tissues, TMZ resistant cells and in glioma stem cells [24–26]. Moreover, their genetic knockdown or pharmacological inhibition impairs glioma cell proliferation and tumor growth, reduces glioma stem cell activity and sensitizes glioma cells to TMZ [24, 26–28]. Moreover, HDAC1 and HDAC6 are, together with HDAC3, the HDACs with lower half maximal effective concentration (EC_50_) for SAHA [29]. Therefore, we selected these 2 HDACs for further characterization and analysis.

Next, we studied the association of selected genes with patient survival in glioblastoma, selecting the best cutoff for each gene in the different cohorts. In this context, we detected differences between the expression of the HDACs, MAOs and ChEs within cohorts, with high expression of HDAC1 and MAO-B associated with worse overall survival in most of the cohorts (Fig. 1D-F and Fig. Suppl. 1B**)**. Moreover, the levels of HDAC6 and BChE were also significantly associated with poor patient outcome in some cases (Fig. 1D-F and Fig. Suppl. 1B**)**.

We next looked for publicly available single-cell RNAseq data to study proteins’ expression in different cell populations. For that purpose, we studied their expression in publicly available datasets from healthy brain tissue and glioblastoma samples. In healthy brain tissue, the expression of HDACs and MAOs is high in neurons and astrocytes, which also present some expression of ChEs. Their expression is also elevated in oligodendrocyte precursor cells (OPCs), showing that target genes are expressed in the cells of origin of glioblastoma **(**Fig. 1G**)**. In the case of glioblastoma samples, the expression of HDACs was observed in multiple cell types, whereas the expression of MAOs and ChEs (mainly MAO-B and BChE) was higher in malignant cells, a population characterized by presenting gain of chromosome 7 and loss of chromosome 10. In addition, their expression is also high in OPCs, which is in line with the healthy brain tissue data **(**Fig. 1H, I and Fig. Suppl. 1C**)**. We also tested the expression of target genes in TCGA cohorts from additional cancers, finding that some HDACs, MAOs and ChEs are increased in stomach, pancreatic and lung adenocarcinoma **(**Fig. Suppl. 2**)**.

### Novel MSM compound – DDI199

**Fig. 2:**
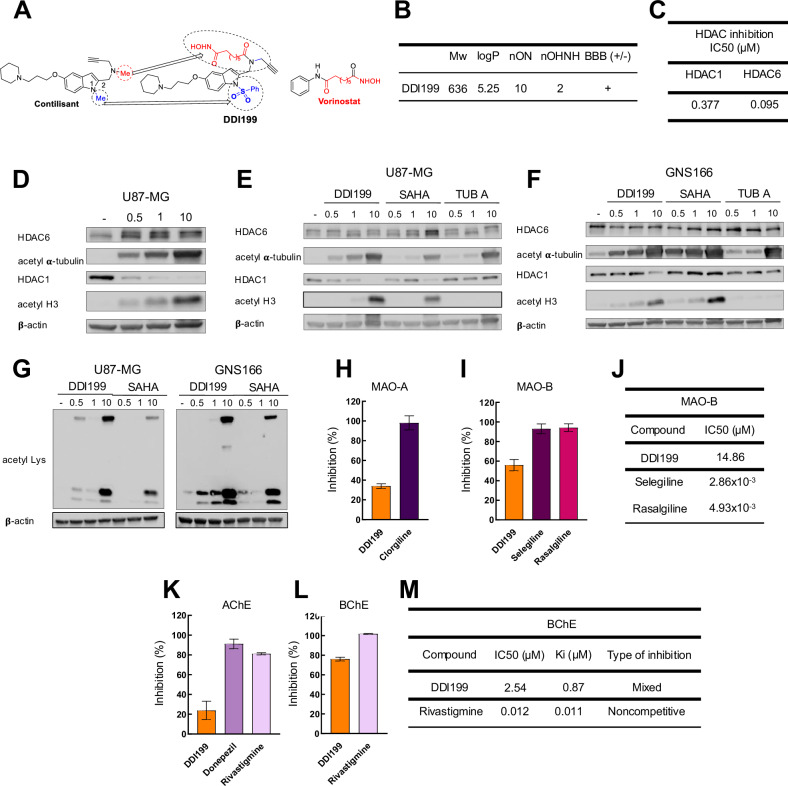
Novel DDI199 compound inhibits target genes. **A** Schematic representation of the structural origin of novel DDI199 compound. **B** Physicochemical characteristics of DDI199. MW: molecular weight (g/mol); logP: log octanol/water partition coefficient; nON: number of Hydrogen acceptors; nOHNH: number of Hydrogen donors; BBB (+/-): blood-brain barrier permeability where + and – represents molecule cross or not the BBB. **C** IC50 values of DDI199 for HDAC1 and HDAC6. **D-F** Representative western blot of HDAC6 and HDAC1 and their targets acetyl α-tubulin and acetyl H3 in **(D-E)** U87-MG and **(F)** GNS166 cells cultured with DDI199, SAHA and Tubastatin A (TUB A) (*n* = 3). **G** Representative western blot of Acetylated-Lysine in U87-MG and GNS166 cells cultured with indicated concentrations of DDI199 and SAHA (*n* = 2). **H**, **I** MAO-A and MAO-B inhibitory capacity of DDI199 and reference compounds Clorgiline Selegiline and Rasalgiline. **J** IC50 values of indicated compounds as MAO-B inhibitors. **K**, **L** AChE and BChE inhibitory capacity of DDI199 and reference compounds Donepezil and Rivastigmine. **M** IC50 values of indicated compounds as BChE inhibitors.

Given the above-described data, we conceived DDI199 as a new Contilisant and SAHA hybrid. DDI199 is a polyfunctionalized indole derivative designed by combining selected pharmacophoric groups from reference ligands Contilisant and SAHA **(**Fig. 2A**)**. Thus, this MSM has been designed to inhibit HDAC, ChE and MAO enzymes, as well as modulate S1R and H3R. Notably, in DDI199 the *methyl* group on the (HC ≡ CCH_2_)NCH_2_*Me* side chain at C(2) of Contilisant (dashed circle (red)), has been substituted with the suberoylanilide hydroxamic acid pharmacophore present in SAHA **(**Fig. 2A**)**. Similarly, the *methyl* group at N(1)*Me* (dashed circle (blue)), of the indole has been replaced by a “*phenylsulfonamide*” group, a well-known 5-HT_6_ receptor (5-HT_6_R) pharmacophore, known to play key roles in cognitive functions [30], and also target the EphA2 receptor with antiproliferative activity on the U251 glioblastoma cell line [31]. Consequently, the new modifications incorporate functional groups to DDI199, designed to enhance its therapeutic potential for the treatment of neurodegenerative diseases and cancer. Detailed information of DDI199 preparation has been described in a preprint [17]. Moreover, the structure of DDI199 has been confirmed by its spectroscopic -IR, ^¡^H NMR, and ^13^C NMR spectra- (Fig. Suppl. 3A-C), and analytical data (HRMS spectrum) (Fig. Suppl. 3D). The purity of DDI199 has been determined by HPLC-MS analysis (Fig. Suppl. 4), confirming that DDI199 MSM is of pharmacological purity for the biological assays.

To further describe the general characteristics of DDI199, online available tools were used. Molinspiration Calculation of Molecular Properties toolkit was used to predict the physicochemical properties of the MSM based on its structure. DDI199 displayed good drug-like structure since it presents logP ≤5, number of hydrogen bond acceptors ≤10 and number of hydrogen bond donors ≤5 **(**Fig. 2B**)**. According to the CBLigand-BBB prediction server, DDI199 would be able to cross the blood-brain barrier (BBB) **(**Fig. 2B**)**. Besides, the toxicity risk was also predicted using OSIRIS Property Explorer, which indicated that DDI199 was not mutagenic, tumorigenic or irritant.

The potential of DDI99 to inhibit relevant targets was then evaluated. First, we studied the effect on HDACs. For that purpose, the IC_50_ for HDAC1 and HDAC6 was calculated, and results showed a higher effect on HDAC6 as the IC_50_ was 0.09 µM for HDAC6 and 0.37 µM for HDAC1 (Fig. 2C). Next, we tested the effect on HDACs protein levels and their targets by western blot in U87-MG. DDI199 did not alter the levels of HDAC6 but promoted a decrease in HDAC1 (Fig. 2D). Noteworthy, their targets acetylated α-tubulin and acetylated H3, respectively, increased their levels with increasing concentrations (0.5, 1 and 10 µM) of DDI199 (Fig. 2D), revealing that it inhibits HDAC6 and HDAC1 activity in a dose-dependent manner. Next, we characterized the effect on patient-derived glioma stem cells directly (GNS166 cells) and compared it to SAHA and Tubastatin A as reference pan-HDAC and HDAC6 inhibitors. Of note, DDI199 promoted higher acetylated α-tubulin and acetylated H3 increase than SAHA and Tubastatin A in both U87-MG and glioma stem cells (Fig. 2E, F). In regard to HDAC6 and HDAC1 expression, the former was not altered, whereas the latter was reduced with the highest concentration of SAHA in U87MG and with DDI199 in GNS (Fig. 2E, F). This effect has been previously observed and it is likely dependent on the targeted region, structure of the inhibitor and cell type [26, 32]. Finally, the treatment of DDI199 and SAHA enhanced lysine acetylation of proteins in a dose-dependent manner, being the effect greater with DDI199 (Fig. 2G), together revealing that it exerts a higher inhibitory capacity on HDACs than reference compounds

Next, the ability of DDI199 to inhibit MAOs and ChEs was tested, for which the inhibition percentage at 10 µM of the compound was measured. Regarding MAOs, we observed that DDI199 displayed a 55% of inhibition MAO-B and 35% in MAO-A (Fig. 2H, I). The IC_50_ was then measured for MAO-B, obtaining a value of 14.86 µM (Fig. 2J). Regarding ChEs, DDI199 was able to inhibit both AChE and BChE, with an inhibition percentage of 25% and 75%, respectively (Fig. 2K, L). In addition, the type of inhibition and constant of inhibition (Ki) for BChE were studied from Lineweaver-Burk and secondary plots. The results showed that DDI199 displayed a Ki of 0.87 µM and, differentially to Rivastigmine reference compound, presented a mixed inhibition, that is, it is able to bind to both the enzyme alone and to the enzyme-substrate complex (Fig. 2M and Fig. Suppl. 5A, B). These results confirm that DDI199 exerts an inhibitory capacity on MAOs and ChEs, especially in MAO-B and BChE.

### DDI199 is cytotoxic for glioma cells and patient-derived stem cells in vitro

**Fig. 3:**
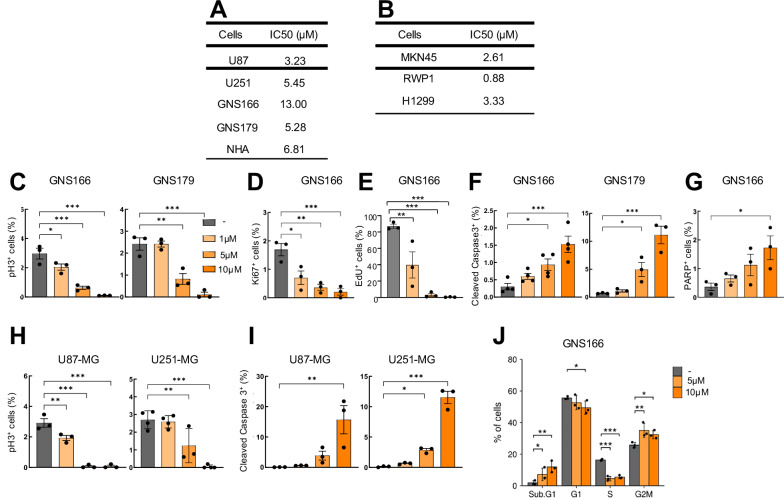
DDI199 is cytotoxic for tumor cells. **A** IC50 values of DDI199 in glioma and patient-derived GSCs as wells as human NHA primary astrocytes measured by MTT. **B** IC50 values of DDI199 in gastric cancer (MKN45), pancreatic cancer (RWP1) and lung cancer (H1299) measured by MTT. **C** Quantification of phospho Histone 3 (pH3) positive cells at 1, 5 and 10 µM concentrations of DDI199 in GNS166 and GNS179 patient-derived GSCs (*n* ≥ 3). **D**, **E** Quantification of Ki67 and EdU positive cells at same concentrations in GNS166 (*n* = 3). **F** Quantification of Caspase3 positive cells at 1, 5 and 10 µM of DDI199 in GNS166 and GNS179 patient-derived GSCs (*n* ≥ 3). **G** Quantification of PARP1 positive cells at same concentrations in GNS166 (*n* = 3). **H**, **I** Quantification of Caspase3 and phospho Histone 3 (pH3) positive cells at indicated concentrations of DDI199 in U87 and U251-MG glioma cells (*n* ≥ 3). **J** Cell cycle distribution in non-treated cells compared to cells treated with 5 and 10 µM of DDI199, assessed by flow cytometry (*n* = 3).

In order to test the potential antitumor effect of DDI199, cell viability was studied in glioma cells and patient-derived GSCs as well as in human NHA primary astrocytes. For this, cells were cultured with increasing doses for 72 hours and IC_50_ values were obtained. The results showed that IC_50_ was lower than 5 µM in different glioma cells whereas was higher in NHA cells (7 µM). Regarding patient-derived GSCs, the IC_50_ ranged between 5 and 13 µM revealing higher heterogeneity but in the range of NHA cells (Fig. 3A). Additional cell viability studies were performed in other types of cancer, showing that the cytotoxicity of DDI199 is not exclusive for glioma cells but it is also applicable to other tested tumor cells including gastric, pancreatic and lung cancer cells. Indeed, the IC_50_ was lower than 3 µM in these contexts (Fig. 3B). These results highlight the antitumor potential of the compound.

To test whether the novel MSM compound could target the population of GSCs, we studied the apoptosis and proliferative capacity of 2 independent patient-derived GSCs (GNS166 and GNS179) treated with increasing concentrations of DDI199 (1, 5 and 10 µM) for 72 hours. In regard to proliferation, we first measured the number of positive cells for the mitosis marker phospho-Histone3 (pH3) by immunofluorescence and detected that the increasing concentrations of DDI199 promoted a marked decrease of pH3-positive cells in both GNS cells (3 and 2.4% in controls to 2 and 2.4% with 1 µM, 0.6 and 0.8% with 5 µM and 0.1 and 0.11% with 10 µM) indicative of a dose-dependent reduction of proliferation (Fig. 3C). These results were further validated with the analysis of Ki67 and EdU positive cells. In both experiments, we detected a dose-dependent decrease on proliferation markers, with 5 µM of DDI199 almost blocking completely the expression of both markers (Fig. 3D, E). For apoptosis, the increasing concentrations of DDI199 promoted an increase of Cleaved Caspase-3-positive cells in both GNS cells (Fig. 3F). Similar results were obtained with PARP-positive cells (Fig. 3G), indicative of a dose-dependent induction of apoptosis. The same immunofluorescences of pH3 and Cleaved Caspase-3 positive were performed in U87-MG and U251-MG cells and similar results were also observed on them. Thus, 1, 5 and 10 μM DDI199 markedly increased and reduced the number of positive cells for Caspase-3 and p-H3, respectively (Fig. 3H, I). Finally, the effect of DDI199 on cell cycle distribution was examined by flow cytometry. GNS166 were cultured with 2 concentrations (5 and 10 µM) and the results showed that DDI199 stimulated the cell subpopulation on Sub G1 (1.9% *vs* 7.2% and 11.9% in increasing concentrations) as well as in G2/M phase (25% *vs* 35 and 32% in treated). Together, these results support that DDI199 significantly inhibits GSC activity.

### DDI199 deregulates pathways related to cell cycle and neurotransmission in GSCs

**Fig. 4:**
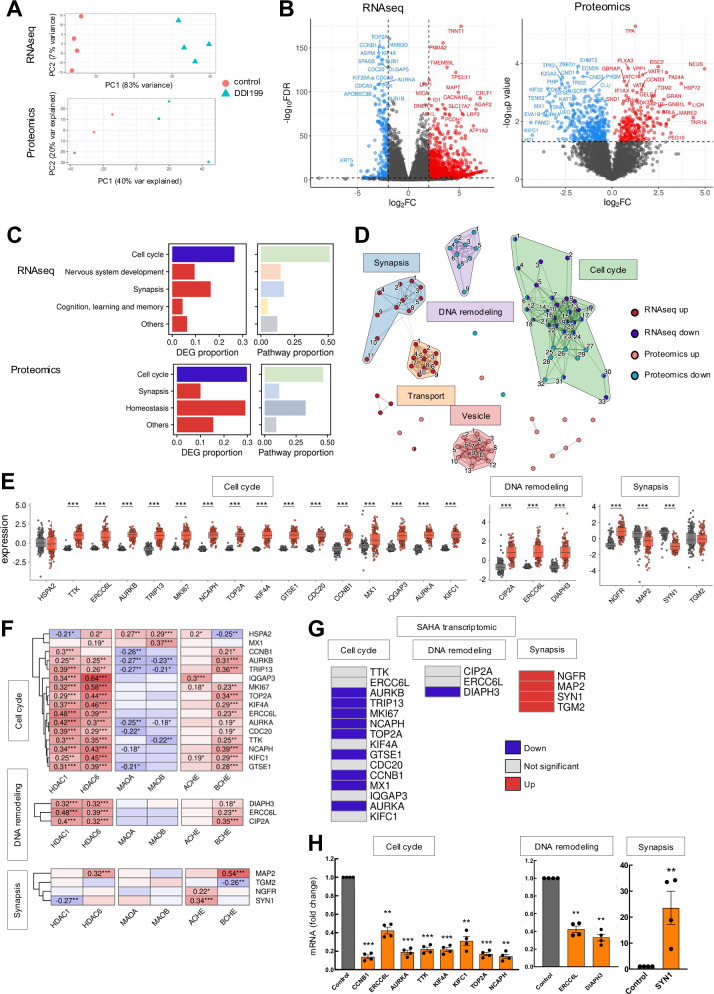
DDI199 treatment deregulates cell cycle and neurotransmission pathways in GSCs. **A** PCA plot of RNAseq and proteomics. **B** Volcano plot of RNAseq and proteomics based on Fold-change>4 and *p*-value < 0.01 for RNAseq and *p*-value < 0.05 for proteomics. **C** Proportion of DEGs and pathways represented by the biological themes in both OMIC approaches**. D** Clustering of top 25 Up and Down enriched pathways in combined analysis of RNAseq and proteomics. The width of the connecting bars is indicative of the number of genes that are shared between the two pathways. The full list of pathways is available in Fig. Suppl 6B. **E** Normal versus tumor expression of common DEGs of the Cell cycle, DNA remodeling and Synapsis clusters in the TCGA cohort. **F** Correlation analysis of common DEGs from selected clusters with target genes in TCGA cohort. **G** Expression of common DEGs from selected clusters in glioblastoma cells in a transcriptomic study of SAHA treatment (GEO accession GSE143887) [27]. **H** q-RTPCR of selected DEGs from each cluster in GNS166 non treated and treated cells (*n* = 4).

To identify the profile of genes whose expression is altered by DDI199 and unravel the molecular mechanisms underlying the activity of the novel MSM compound, RNAseq and proteomic studies were performed in GNS166 cells non-treated and treated with 5 µM of DDI199. Overall, PCA presented clear differences between control nontreated and treatment group (Fig. 4A), obtaining a large number of differentially expressed genes using a fold-change cutoff > 4 and *p* value < 0.01 for RNAseq and *p* value < 0.05 for proteomics. In particular, a total of 952 upregulated and 204 downregulated genes were obtained in the RNAseq study, while 217 upregulated and 391 downregulated proteins were obtained in proteomics (Fig. 4B and Table Suppl 1). Gene Ontology analysis in the RNAseq study revealed that the most differentially genes and enriched pathways associated with downregulated genes were linked to cell cycle (Fig. 4C and Fig. Suppl 6A). The same analysis in the proteomic study showed similar results with most of the downregulated genes and pathways also related to cell cycle (Fig. 4C and Fig. Suppl 6B). However, in this case, epigenetic regulation of gene expression was altered as well (Fig. Suppl 6B). The cell cycle reduction is coherent with the functional results of proliferation described in Fig. 3 and the reduction in epigenetic regulation might be associated with the effect of DDI199 on HDACs. Gene Ontology analysis also revealed similar results in both OMIC approaches with respect to upregulated genes. In this case, pathways related to neurotransmission/synapsis were detected (Fig. 4C and Fig. Suppl. 6A, B).

With the aim of further characterizing the effect of DDI199 and extend the association between both OMIC approaches, we clustered the top 25 differentially enriched pathways of the four comparisons together (up and down for RNA seq and up and down for proteomic) based on the Jaccard similarity. This analysis revealed five different clusters, being three of them the largest and most significant. These include two downregulated as cell cycle and DNA remodeling and a cluster upregulated, which is related to neurotransmission/synapsis (Fig. 4D and Fig. Suppl. 6C).

Next, we selected the set of differentially expressed genes for each of the 3 clusters. For that, the set of differentially expressed genes in RNAseq and proteomics was taken, and those that were significantly altered in both approaches were selected for further validations. First, we moved back to TCGA samples and study their expression, where we detected that all genes except one from the cell cycle and DNA remodeling clusters presented increased levels in glioblastoma samples compared to control tissue (Fig. 4E). Next, we characterized their correlation with the main DDI199 targets. Noteworthy, we observed that genes associated with cell cycle and DNA remodeling were positively and significantly correlated with HDACs and BChE and not with MAOs (Fig. 4F). This might indicate that the effect of DDI199 might be more driven by HDACs and BChE than MAOs. In order to support this idea, we analyzed the expression of the set of differentially expressed genes on a SAHA treatment transcriptomic analysis we performed previously in GNS [27]. Indeed, most of the genes related to the 3 clusters were also differentially expressed in SAHA treatment study and showing the same trend, decreased for cell cycle and DNA remodeling and increased for synapsis (Fig. 4G**)**. Finally, the genes of the correlation study were selected for their validation by q-RTPCR. The expression of all tested genes associated with cell cycle and DNA remodeling was significantly reduced in DDI199 treated compared to non-treated cells, while the expression of the associated to neurotransmission/synapsis was significantly higher (Fig. 4H).

### DDI199 reduces GBM tumorigenicity in vivo

**Fig. 5:**
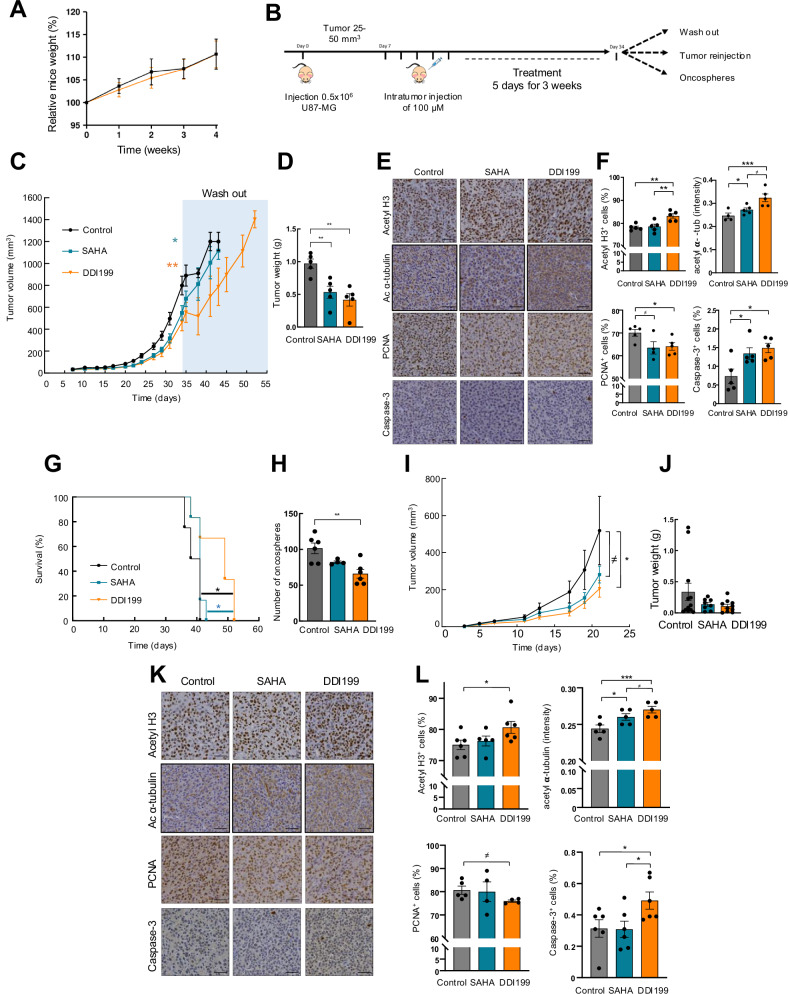
DDI199 reduces tumor growth in vivo. **A** Relative change in body weight of mice treated with vehicle (black) and treated with 15 mg/Kg of DDI199 (orange) (*n* = 5). **B** Schematic representation of the experiment workflow. **C** Measurement of tumor volume at indicated timepoints of mice treated intratumorally with vehicle (control), or 100 µM of SAHA or DDI199 (*n* ≥ 13) and washout (*n* ≥ 4). **D** Tumor weight at end-point day 34 (*n* = 5). **E**, **F** Representative Immunohistochemistry and quantification of acetyl H3, acetyl α-tubulin, PCNA and Caspase-3 at end-point day 34 (*n* ≥ 4). Scale bar represents 50 µm. **G** Survival of animals from different conditions in washout experiment (*n* ≥ 4). **H** Quantification of oncospheres derived from tumors of different conditions (*n* = 3 independent tumors and duplicates of each tumor. **I**, **J** Measurement of tumor volume and weight of mice re-injected with cells isolated from end-point at day 34 (*n* ≥ 8). **K**, **L** Representative Immunohistochemistry and quantification of acetyl H3, acetyl α-tubulin, PCNA and Caspase-3 of re-injection experiment (*n* ≥ 4). Scale bar represents 50 µm.

We first tested the cytotoxicity of DDI199 in vivo, and performed a study injecting intraperitoneally 15 mg/mL of DDI199 5 times per week for 4 weeks. The effects of DDI199 were evaluated on body weight and organ macroscopic analysis. The treatment during the entire experiment did not significantly affect the body weight of animals compared to controls (Fig. 5A). Moreover, we did not observe any difference between the different organs among the experimental groups in the macroscopic analysis of the animals at the end-point.

To evaluate DDI199 antitumorigenic potential in vivo, we first characterized its effect in single-treatment experiments. For that, U87-MG cells were injected subcutaneously in immunodeficient mice and once tumors were, they were treated with vehicle and DDI199 or SAHA for 4 weeks intratumorally (Fig. 5B). Of note, DDI199 significantly reduced the volume of tumors formed in control animals since the first treatments. At the endpoint of 34 days, tumors treated with DDI199 reached a mean final volume of 465 mm^3^ compared to 800 mm^3^ in controls (Fig. 5C), supposing a reduction of almost 50% in tumor growth. Moreover, the comparison of DDI199 with SAHA treatment showed that DDI199 exerts a higher anti-tumor activity since SAHA tumors reached a final volume of 550 mm^3^, although this difference was not statistically different (Fig. 5C). Consistently, the weight of the tumors at the end-point was significantly lower than the controls and also of SAHA (Fig. 5D and Fig. Suppl 7A). In addition, immunohistochemical stainings showed that tumors from DDI199 treated condition presented greater acetyl H3 (78% *vs* 83%) and acetyl **α**-tubulin (0.32 *vs* 0.25) immunohistochemical staining, significantly lower positive cells for PCNA proliferation marker (64% *vs* 70%) and higher Cleaved Caspase 3 positive cells (1.5% *vs* 0.7%) compared to control ones (Fig. 5E, F). which validates the in vivo activity of the novel MSM compound. Moreover, the effect of SAHA treatment in these markers tended to be lower than DDI199, although only H3 acetylation showed significant differences (Fig. 5E, F).

In order to study the long-term effect of DDI199, we completed 2 independent experiments. On one hand, some of the mice (*n* = 4) treated with vehicle and DDI199 or SAHA for 4 weeks were maintained without treatment (washout experiment) in which mice were sacrificed when they reached end-point volume. Notably, we found that DDI199 treated animals displayed reduced tumor volume and significantly longer survival compared to controls and SAHA (*p* = 0.05) (Fig. 5C, G). In detail, controls and SAHA treated reached the end-point volume 10 days after washout experiments compared to 20 days in DDI199 group. On the other hand, resected tumors treated with vehicle, DDI199 or SAHA were disaggregated and further experiments of stem cell activity and tumor re-growth were performed. In the former experiment, tumor cells were cultured in GSC medium for 7 days and the number of oncospheres was counted. Cells from DDI199 treated tumors formed fewer oncospheres (Fig. 5H). On the later, tumor cells were subcutaneously re-injected in immunodeficient mice and tumor growth was followed. Cells derived from DDI199 treated tumors grew significantly lower compared to non-treated control cells (Fig. 5I). In detail, tumors derived from control cells reached a mean volume of over 500 mm^3^ compared to 200 mm^3^ in tumors from DDI199 cells (Fig. 5I). These results show a reduction of 60%, which was also reflected in the final tumor weight, with a reduction of almost 70% (Fig. 5J and Fig. Suppl 7B). Immunohistochemical stainings confirmed that the antitumorigenic activity of DDI199 is maintained for long period in vivo since the number of PCNA and Cleaved Caspase 3 positive cells were lower and higher, respectively, in DDI199 tumors compared to controls (Fig. 5K, L). The inhibitory effect on targets was also maintained as acetyl H3 expression and acetyl **α**-tubulin was higher in DDI199 tumors (Fig. 5K, L). In all these experiments, especially in acetyl **α**-tubulin and Cleaved Caspase 3, the effect of DDI199 tended to be higher when compared with SAHA treatment.

### Combined therapy of DDI199 with TMZ chemotherapy presents a synergistic effect in vivo

**Fig. 6:**
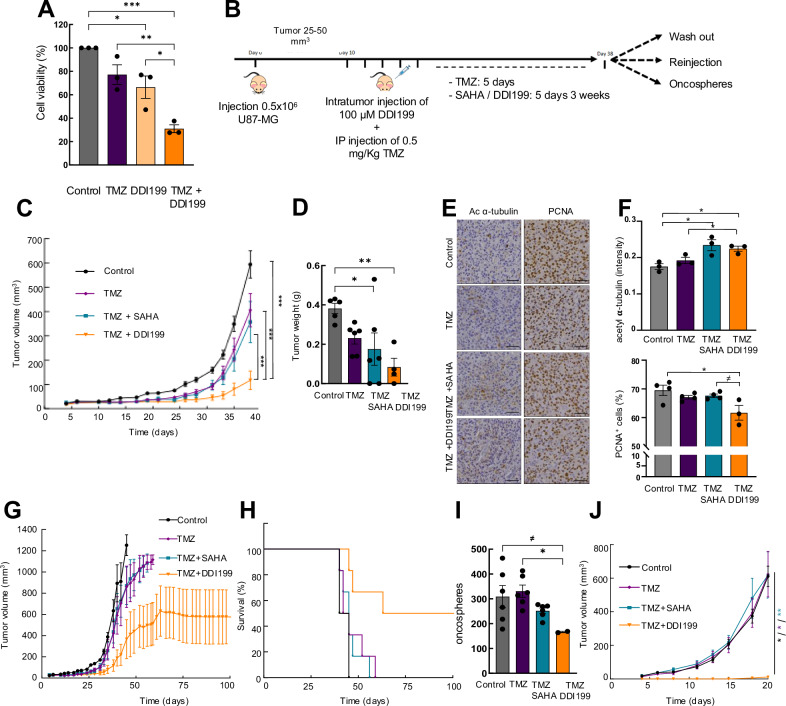
Combined treatment of DDI199 and TMZ display potent anti-tumor activity. **A** Study of cell viability of U87-MG cells treated with TMZ and DDI199 alone or combined treatment for 72 hours (*n* = 3). **B** Schematic representation of in vivo experiment workflow. **C** Measurement of tumor volume at indicated timepoints of mice treated intraperitoneally with vehicle or TMZ 0.5 mg/Kg and intratumorally with SAHA or DDI199 100 µM (*n* ≥ 10). **D** Tumor weight end-point day (*n* ≥ 4). **E**, **F** Representative Immunohistochemistry and quantification of acetyl α-tubulin and PCNA at end-point day 38 (*n* ≥ 3). Scale bar represents 50 µm. **G** Tumor volume of mice from washout experiment. **H** Survival plot of mice from washout experiment (*n* ≥ 4). **I** Quantification of oncospheres derived from tumors of different conditions (*n* ≥ 2 independent tumors). **J** Measurement of tumor volume of mice re-injected with cells isolated from end-point at day 36 (*n* ≥ 3).

Taking into account the promising results obtained in single-treatment in vitro and in vivo experiments, we next studied if DDI199 could be complementary and/or additive to TMZ activity in combination. For this, we first performed cell viability experiments in vitro. We detected that 250 µM of TMZ and 0.5 µM of DDI199 reduced cell viability compared to non-treated cells. Notably, a synergistic effect was observed when both compounds were used in combination with over 70% cytotoxicity in glioma cells (Fig. 6A). Next, we moved to the in vivo setting and completed a similar experiment as in the single-treatment approach (Fig. 6B). For this, U87-MG cells were injected subcutaneously in immunodeficient mice and when the tumor was detected in the range of 25-50 mm^3^ of volume, they were treated with vehicle or 0.5 mg/kg of TMZ during 5 days intraperitoneally and with DDI199 or SAHA during 3 weeks intratumorally. As expected, TMZ treatment reduced tumor growth of glioma cells (Fig. 6C). Noteworthy, the addition of DDI199 or SAHA further reduced tumor volume, however, the reduction was significantly more remarkable in the case of the DDI199 (Fig. 6C). In particular, combined treatment of TMZ and DDI199 reached a mean volume of 115 mm^3^ compared to 600 mm^3^ in controls and 355 mm^3^ in SAHA, achieving a reduction of 80% and almost 70% in final tumor volume, confirmed by a reduction of 65% and 47% in tumor weight (Fig. 6C, D and Fig. Suppl 7C). TMZ treatment alone reached a mean volume of 400mm^3^, and thus, addition of DDI199 reduced 70% tumor volume and 65% tumor weight (Fig. 6C, D and Fig. Suppl 7C). In line with these results tumors from DDI199 treated condition presented significantly greater acetyl-α-tubulin immunohistochemical staining and less positive cells for PCNA compared to non-treated ones (Fig. 6E, F**)**. Of note, some of the tumors treated with the combination of TMZ + DDI199 disappeared or completely stopped their growth compared to the TMZ alone group, reinforcing the complementary antitumor effect of the combined treatment.

In this context, we also performed the 2 independent experiments cited above to study the long-term effect of the combination of TMZ plus DDI199. Regarding washout experiment, tumors derived from combined treatment with TMZ and DDI199 presented significantly reduced tumor volume and presented extended survival compared to the other 3 groups (control, TMZ alone and TMZ plus SAHA (Fig. 6G, H). Similarly, disaggregated cells derived from resected tumors TMZ plus DDI199 formed significantly lower number of oncospheres than control and TMZ-treated cells (Fig. 6I). Moreover, and with respect to the regrowth experiment, no differences were detected between control, TMZ and TMZ + SAHA group in tumor volume and tumor weight (Fig. 6J and Fig. Suppl 7D). However, the differences with TMZ + DDI199 derived cells were massive with only 1 tumor out of 3 reforming tumors and these were significantly lower when compared with the rest of the groups (Fig. 6J and Fig. Suppl 7D).

## Discussion

MSMs are gaining significant interest for the treatment of multifactorial diseases in recent years, including several types of cancer [15], because they enable to target the high heterogeneity that characterizes these diseases. Indeed, intra and inter-tumor heterogeneity are the main limitations for current therapy treatments, that leads to recurrence and therapy resistance [13, 14].

In this work, we characterized and validated the anti-tumor activity of a novel multi-target molecule entitled DDI199 or contilistat [17]. This compound combines AChE/BChE and MAO A/B inhibition with histamine H3 receptor (H_3_R)/sigma 1 receptor (S_1_R) modulation [4] with the incorporation of pharmacophoric groups similar to the HDAC pan-inhibitor SAHA. First, and in accordance with previous studies [27, 33–36], we observed that the expression of different HDACs, MAOs and ChEs, especially HDAC1, MAO-B and BChE were increased in glioblastoma samples from different cohorts and its high levels associated with poor patient survival. Together, these results support that inhibition of targets of the novel MSM might be a promising strategy for glioblastoma therapy.

Current therapy of radiotherapy and chemotherapy with TMZ displays partial effectiveness as they eliminate proliferative cells but have little effect in GSC subpopulation, which are maintained mostly in quiescence. Moreover, this population is also responsible for tumor recurrence as they display self-renewal activity when activated [37]. Of note, we detected that target genes were highly expressed in astrocytes, OPCs and in malignant cells, in particular, in single-cell RNAseq studies of healthy brain tissue and glioblastoma samples. Thus, these results support the relevance of target genes in glioblastoma cell heterogeneity and reinforce the idea that the inhibition of these targets with a multi-target compound is a promising strategy for the treatment of glioblastoma. In this line, previous studies have shown that pan-HDAC inhibitors, including SAHA, as well as specific inhibitors for HDAC1 or HDAC6 [27, 38, 39] inhibit the activity of GSCs. Noteworthy, in this work we revealed that DDI199 is able to inhibit HDAC activity with a greater intensity than SAHA and that it seems more specific to HDAC6 than to HDAC1, being the expression of HDAC6 enriched in GSCs [27]. Consistent with this, we found that DDI199 is cytotoxic for the subpopulation GSCs as it induces their apoptosis while reducing their proliferation. Moreover, DDI199 also presents MAO and ChE inhibitory activity validating the multitarget activity. As a limitation of our study, we did not characterize the effect on the histamine H3 receptor and sigma 1 receptor. However, previous studies revealed the effectiveness of inhibiting their expression or activity [40, 41], and it is expected that part of the anti-tumor activity of DDI199 are due to these targets. This idea is in line with recent studies of additional multi-target compounds sharing some of the targets of DDI199. In one of such studies, the effect of novel compounds against glioblastoma by inhibiting AChE and modulating S1R and HDACs was observed, while another study showed promising results with dual MAO-A and HDAC inhibitors in glioma [20, 21]. Moreover, it is in line with the OMIC analyses that showed that treatment of GSCs with DDI199 deregulates genes related to neurotransmission/synapsis processes as well as inhibits genes linked to cell cycle and DNA remodeling. The effects on cell cycle are a major characteristic of HDAC inhibition. In this line, a previous study of the group also showed that SAHA promoted the downregulation of pathways related to cell cycle and cellular division, while upregulated genes were related to cell differentiation and death [27]. Besides, previous studies have shown that HDAC inhibitors modulate the expression of cell cycle genes such as cyclin D1, CDKs, INK4a/ARF locus, p21^CIP^, Ki67 or Aurora kinase [42, 43], the latter genes being 2 of the most significantly downregulated gene in MSM treated conditions. Similarly, DNA remodeling and DNA repair genes have been also associated to HDAC inhibition [27, 44]. These results allow us to hypothesize that downregulation of genes associated to cell cycle and DNA remodeling might be mainly due to the inhibition of MSMs on HDACs.

Notably, DDI199 promoted promising results in in vivo experiments, with a significant reduction in tumor growth rate when administered alone and especially in combination with TMZ. Indeed, combined treatment of DDI199 with TMZ promoted a synergistic effect, which was significantly higher than the one produced by SAHA. As a limitation of our study, the in vivo experiments of tumorigenesis were performed in an immunodeficient mouse model with subcutaneous injection U87-MG cells. Additional cell models, injections directly in the brain or the use of immunocompetent mouse models would have provided more relevant models. Online available tools showed that DDI199 could cross the BBB, which is one of the limitations when generating new drugs against glioblastoma and would not be mutagenic, tumorigenic or irritant. Consistent with this, no toxic effects or weight alterations were observed in the animals in the in vivo studies. In this regard, this was an objective in the original structure of DDI199, which is a derivative of Contilisant, and this molecule or additional derivates did not present toxic effects in in vivo studies [3, 4]. This idea, together with the results that the cytotoxicity of DDI199 is higher in glioma cells than primary astrocytes suggests that it presents a promising safety profile in vivo, although complementary studies are likely to be completed.

In summary, in this work we described a novel multi-target compound named DDI199 directed against HDACs, MAOs, ChEs as well as histamine H3 and sigma 1 receptors. We detected that the target specificity and antitumor activity of the novel compound is higher than SAHA pan-HDAC inhibitor. We revealed that it significantly inhibits glioma cell growth and GSC activity and causes programmed cell death in vitro and in vivo. Finally, it exerts a synergistic effect with TMZ showing promising results in the pre-clinical setting.

## Supplementary information

Supplementary Figures

Supplementary data

Original Data

## Data Availability

The transcriptomic data generated in this publication have been deposited in NCBI’s Gene Expression Omnibus and are accessible in GSE282561.The mass spectrometry proteomics data have been deposited to the ProteomeXchange Consortium via the PRIDE partner repository with the dataset identifier PXD058898.
