# Supplementary material for: Generation and validation of a novel multitarget small molecule in glioblastoma
Source: Cell Death Dis. 2025 Apr 4;16(1):250. doi: 10.1038/s41419-025-07569-1 (PMC11971462; doi:10.1038/s41419-025-07569-1)
Supplement: Supplementary file 1 — Supplementary Figures [file 41419_2025_7569_MOESM1_ESM.pdf]

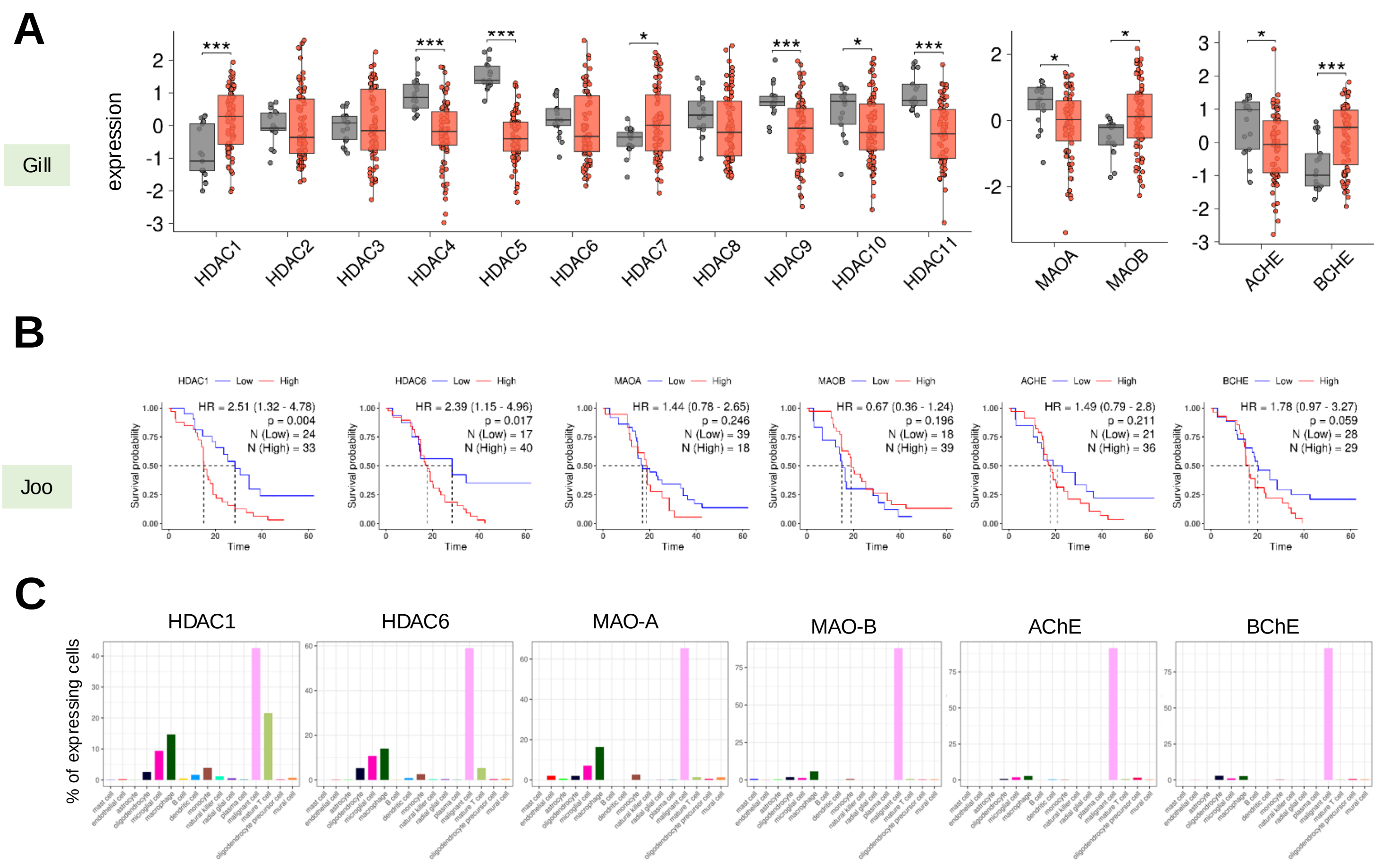

Suppl. Fig. 1. A) Expression of target genes in normal tissue (grey) versus glioblastoma samples (red) in Gill cohort. **B)** Kaplan-Meier survival curves of patients with low vs highEx expression of target genes in Joo cohort. **C)** Expression and percentage of expressing cells of target genes in cell types from single-cell RNAseq of glioblastoma samples.

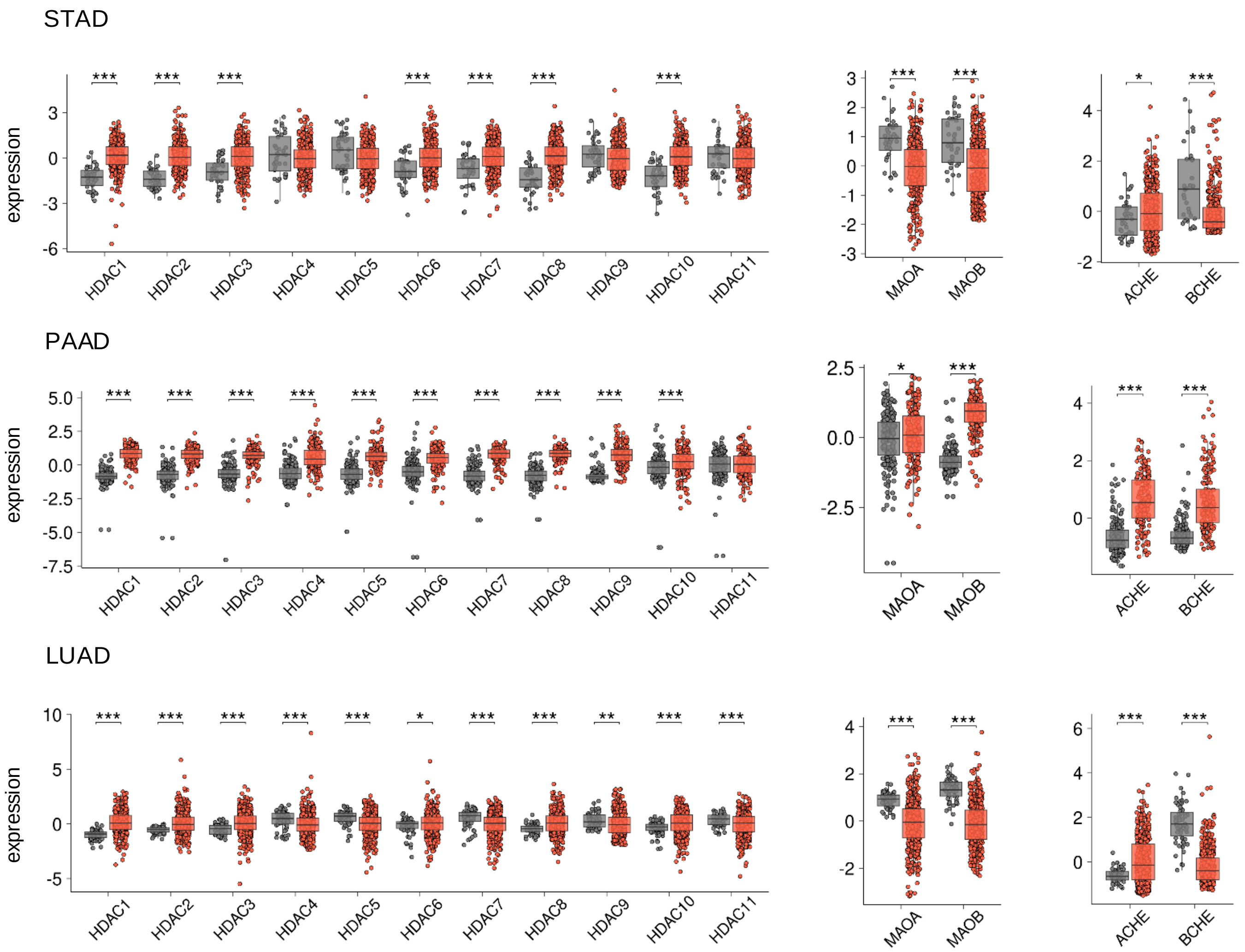

Suppl. Fig. 2. Expression of target genes in normal tissue (grey) versus cancer samples (red) in gastric (STAD), pancreatic (PAAD) and lung (LUAD) cancer in TCGA cohort.

A

IR spectrum

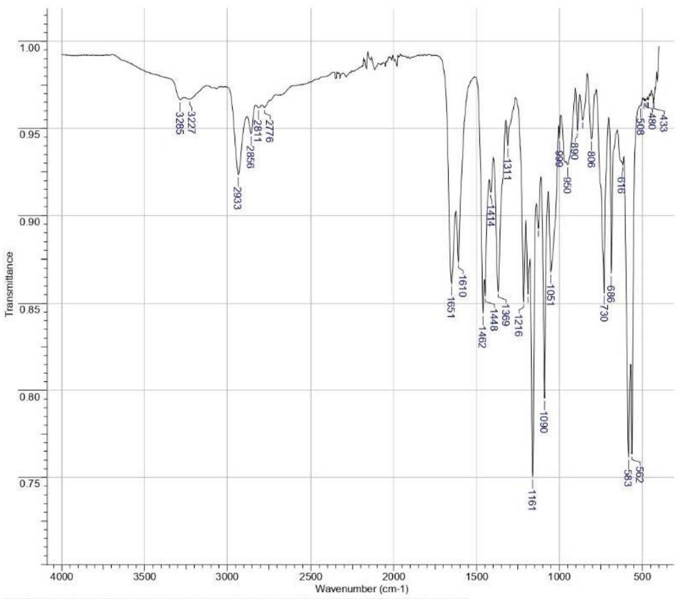

No	cm-1	T	FWHH	Asym	Intensity
1	428.00	0.967	-	-	W
2	433.00	0.965	-	-	W
3	464.00	0.966	-	-	W
4	471.00	0.967	-	-	W
5	480.00	0.966	-	-	W
6	488.00	0.966	-	-	W
7	508.00	0.963	-	-	W
8	562.00	0.763	-	-	VS
9	583.00	0.761	-	-	VS
10	616.00	0.929	-	-	W
11	686.00	0.867	-1.00	0.00	M
12	730.00	0.856	-	-	M
13	806.00	0.944	-1.00	0.00	W
14	859.00	0.955	-	-	W
15	890.00	0.949	-	-	W
16	950.00	0.929	-	-	W
17	999.00	0.944	-	-	W
18	1051.00	0.868	-	-	M

No	cm-1	T	FWHH	Asym	Intensity
19	1090.00	0.795	-1.00	0.00	S
20	1127.00	0.893	-	-	M
21	1161.00	0.750	-1.00	0.00	VS
22	1190.00	0.855	-	-	M
23	1216.00	0.851	-	-	M
24	1311.00	0.940	-	-	W
25	1369.00	0.857	-1.00	0.00	M
26	1414.00	0.913	-	-	M
27	1448.00	0.854	-	-	M
28	1462.00	0.844	-	-	S
29	1610.00	0.874	-	-	M
30	1651.00	0.861	-	-	M
31	2776.00	0.962	-	-	W
32	2811.00	0.962	-	-	W
33	2856.00	0.947	-	-	W
34	2933.00	0.924	-	-	W
35	3227.00	0.967	-	-	W
36	3285.00	0.966	-	-	W

B

^{1}H NMR (100 ºC) spectrum

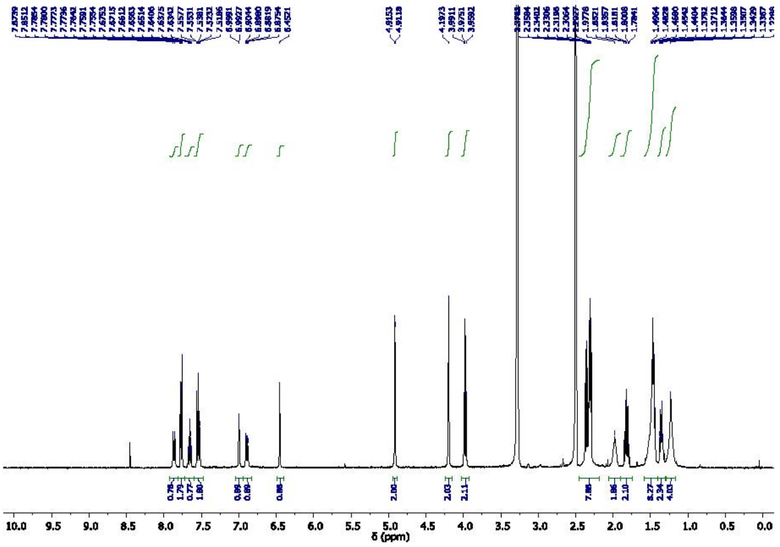

C

^{13}C NMR spectrum

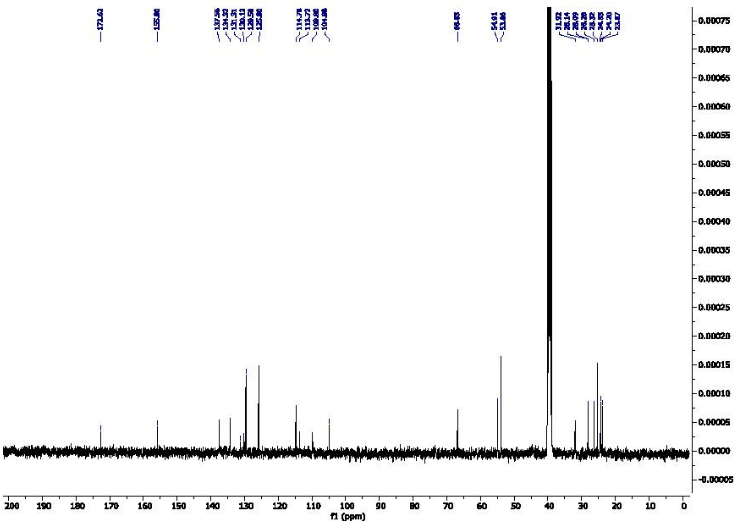

D

HRMS spectrum

Compound Table

Compound Label	RT	Mass	Abund	Formula	Tgt Mass	Diff (ppm)	Hits (DB)
Cpd 1: C34 H44 N4 O6 S; 2.129	2.129	636.2974	1934371	C34 H44 N4 O6 S	636.2982	-1.12	1

Compound Label	*m/z*	RT	Algorithm	Mass
Cpd 1: C34 H44 N4 O6 S; 2.129	637.3045	2.129	Find by Formula	636.2974

MS Zoomed Spectrum

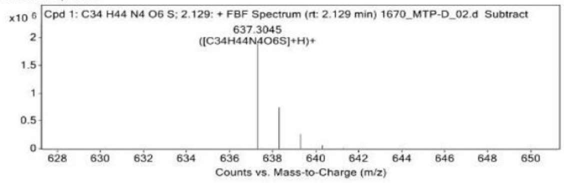

MS Spectrum Peak List

m/z	z	Abund	Formula	Ion
637.3045	1	1934370.88	C34H44N4O6S	(M+H)+
638.3082	1	744057.25	C34H44N4O6S	(M+H)+
639.3074	1	259578.28	C34H44N4O6S	(M+H)+
640.3084	1	60391.55	C34H44N4O6S	(M+H)+
641.3108	1	11383.89	C34H44N4O6S	(M+H)+

MS Zoomed Spectrum

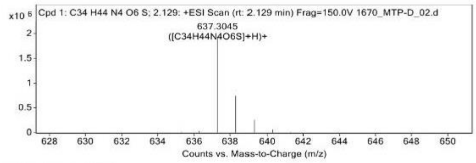

MS Spectrum Peak List

m/z	*Calc m/z*	Diff(ppm)	z	Abund	Formula	Ion
637.3045	637.3054	-1.4	1	1934370.88	C34H44N4O6S	(M+H)+
638.3082	638.3085	-0.53	1	744057.25	C34H44N4O6S	(M+H)+
639.3074	639.308	-0.85	1	259578.28	C34H44N4O6S	(M+H)+
640.3084	640.3088	-0.61	1	60391.55	C34H44N4O6S	(M+H)+
641.3108	641.3102	0.94	1	11383.89	C34H44N4O6S	(M+H)+

Suppl. Fig. 3. Structure of DDI199 molecule based on spectroscopic and analytical data. Analysis with **A)** Infrared spectroscopy (IR), **B)** Proton nuclear magnetic resonance (^{1}H NMR), **C)** Carbon-13 nuclear magnetic resonance (^{13}C NMR), and **D)** High resolution mass spectrometry (HRMS).

HPLC-MS

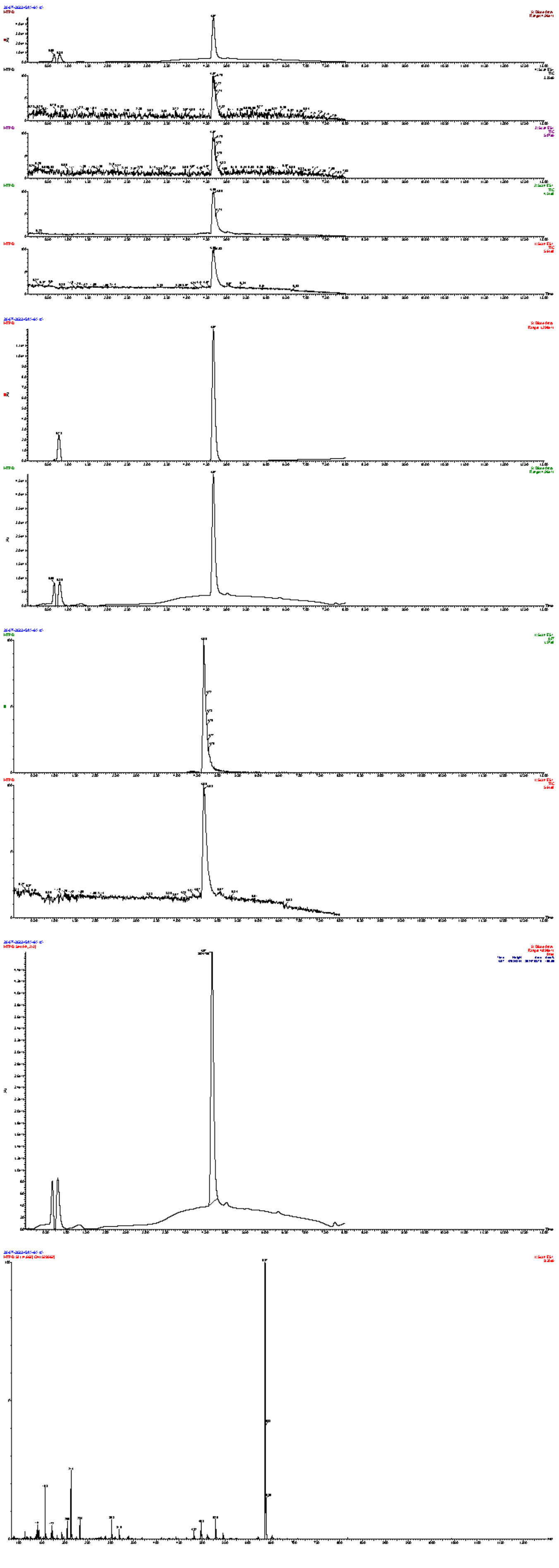

Suppl. Fig. 4. Purity of DDI199. Analysis with High-performance liquid chromatograph with mass spectrometry (HPLC-MS).

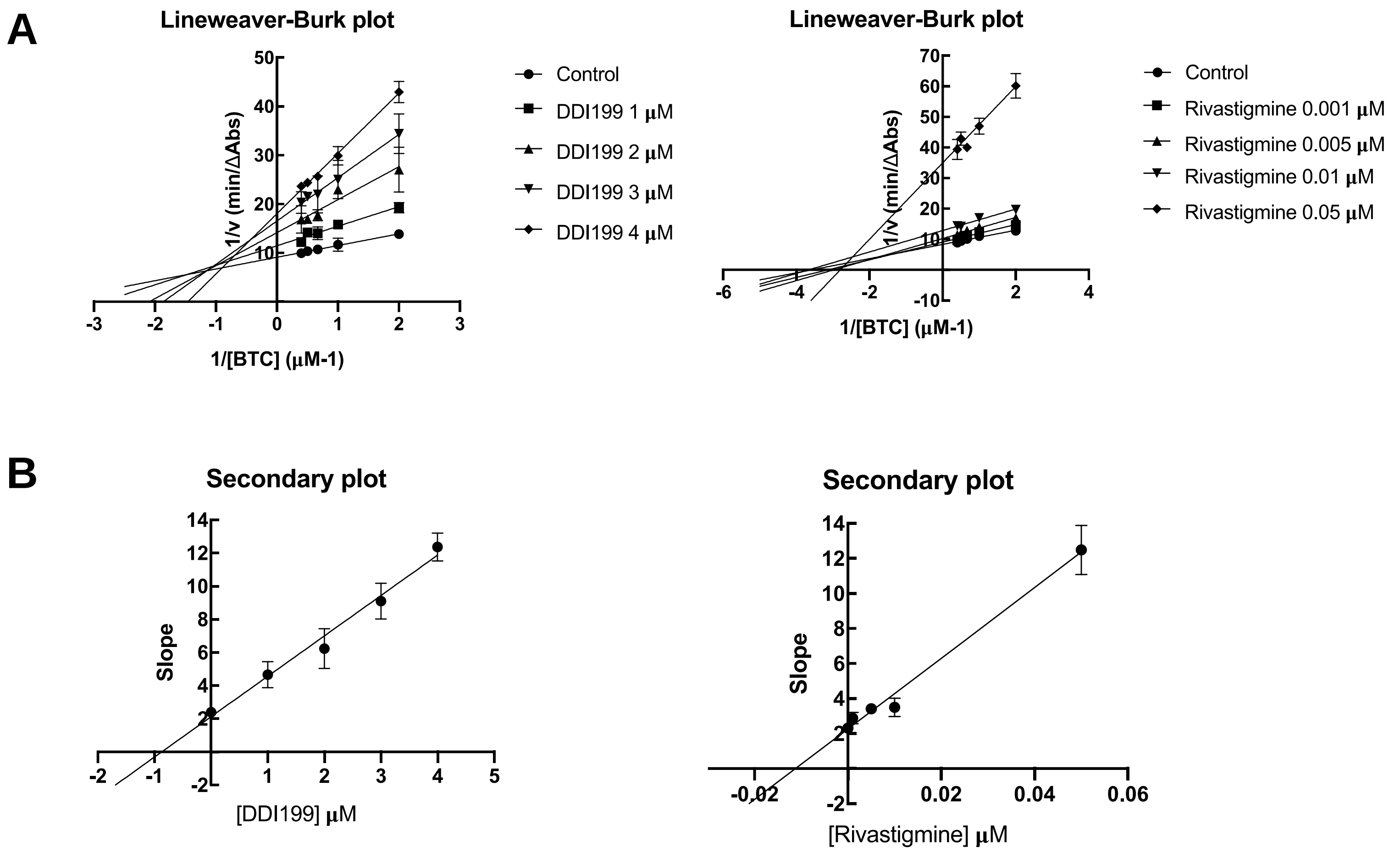

Suppl. Fig. 5 A) Lineweaver-Burk plot representing reciprocal of BChE velocity versus reciprocal of different substrate concentrations at different concentrations of DDI199 or Rivastigmine. **B)** Secondary plot of slopes obtained in Lineweaver-Burk plot versus different inhibitor concentrations for the estimation of Ki for DDI199 and Rivastigmine.

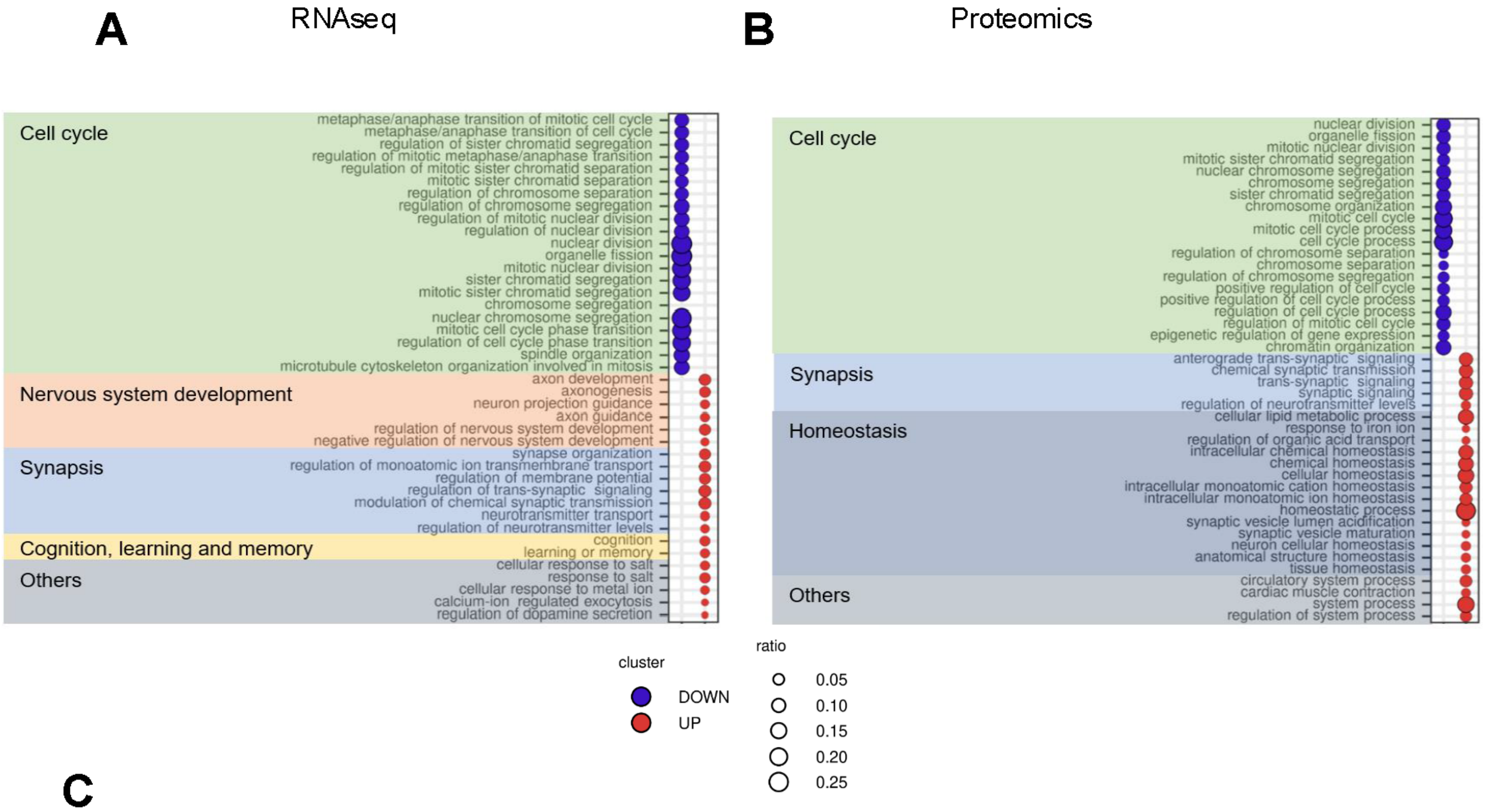

C

	Cell cycle
1	ATP hydrolysis activity
2	cell cycle G2/M phase transition
3	mitotic cytokinesis
4	microtubule motor activity
5	midbody
6	microtubule binding
7	chromosome localization
8	regulation of cell cycle phase transition
9	chromosome, centromeric region
10	mitotic nuclear division
11	meiotic chromosome segregation
12	non-membrane-bounded organelle assembly
13	negative regulation of chromosome organization
14	spindle organization
15	spindle assembly checkpoint signaling
16	nuclear division
17	mitotic spindle checkpoint signaling
18	spindle
19	regulation of mitotic nuclear division
20	regulation of nuclear division
21	chromosome segregation
22	nuclear chromosome segregation
23	regulation of chromosome segregation
24	regulation of chromosome separation
25	sister chromatid segregation
26	regulation of chromosome organization
27	mitotic cell cycle phase transition
28	condensed chromosome
29	regulation of mitotic cell cycle phase transition
30	kinetochore organization
31	chromosome condensation
32	nuclear chromosome
33	centromere complex assembly

	DNA remodeling
1	cadherin binding
2	nucleosome organization
3	nucleosome assembly
4	protein-DNA complex assembly
5	nucleosome
6	protein-DNA complex organization
7	epigenetic regulation of gene expression
8	chromatin remodeling
9	SWI/SNF superfamily-type complex

	Vesicle
1	extrinsic component of synaptic vesicle membrane
2	ATPase-coupled ion transmembrane transporter activity
3	diphosphate hydrolysis-driven proton transmembrane transporter activity
4	proton-transporting V-type ATPase, V1 domain
5	synaptic vesicle maturation
6	neuron cellular homeostasis
7	vacuolar proton-transporting V-type ATPase, V1 domain
8	proton-transporting ATPase activity, rotational mechanism
9	clathrin-coated vesicle membrane
10	synaptic vesicle lumen acidification
11	clathrin-coated vesicle
12	ATPase activity, coupled to transmembrane movement of ions, rotational mechanism
13	macroautophagy

	Synapsis
1	neuron to neuron synapse
2	glutamatergic synapse
3	postsynaptic specialization
4	neurotransmitter transport
5	synaptic membrane
6	modulation of chemical synaptic transmission
7	regulation of trans-synaptic signaling
8	synapse organization
9	cognition
10	regulation of nervous system development
11	axon development

	Transport
1	regulation of membrane potential
2	transporter complex
3	regulation of monoatomic ion transmembrane transport
4	monoatomic ion channel complex
5	voltage-gated monoatomic ion channel activity
6	metal ion transmembrane transporter activity
7	monoatomic ion gated channel activity
8	monoatomic ion channel activity
9	gated channel activity
10	voltage-gated channel activity

Suppl. Fig. 6 A-B) Gene ontology study of RNAseq and proteomics representing the 20 most correlated pathways with upregulated and downregulated DEGs. **C)** List of ontologies conforming each of the clusters from the combined study of RNAseq and proteomic results.

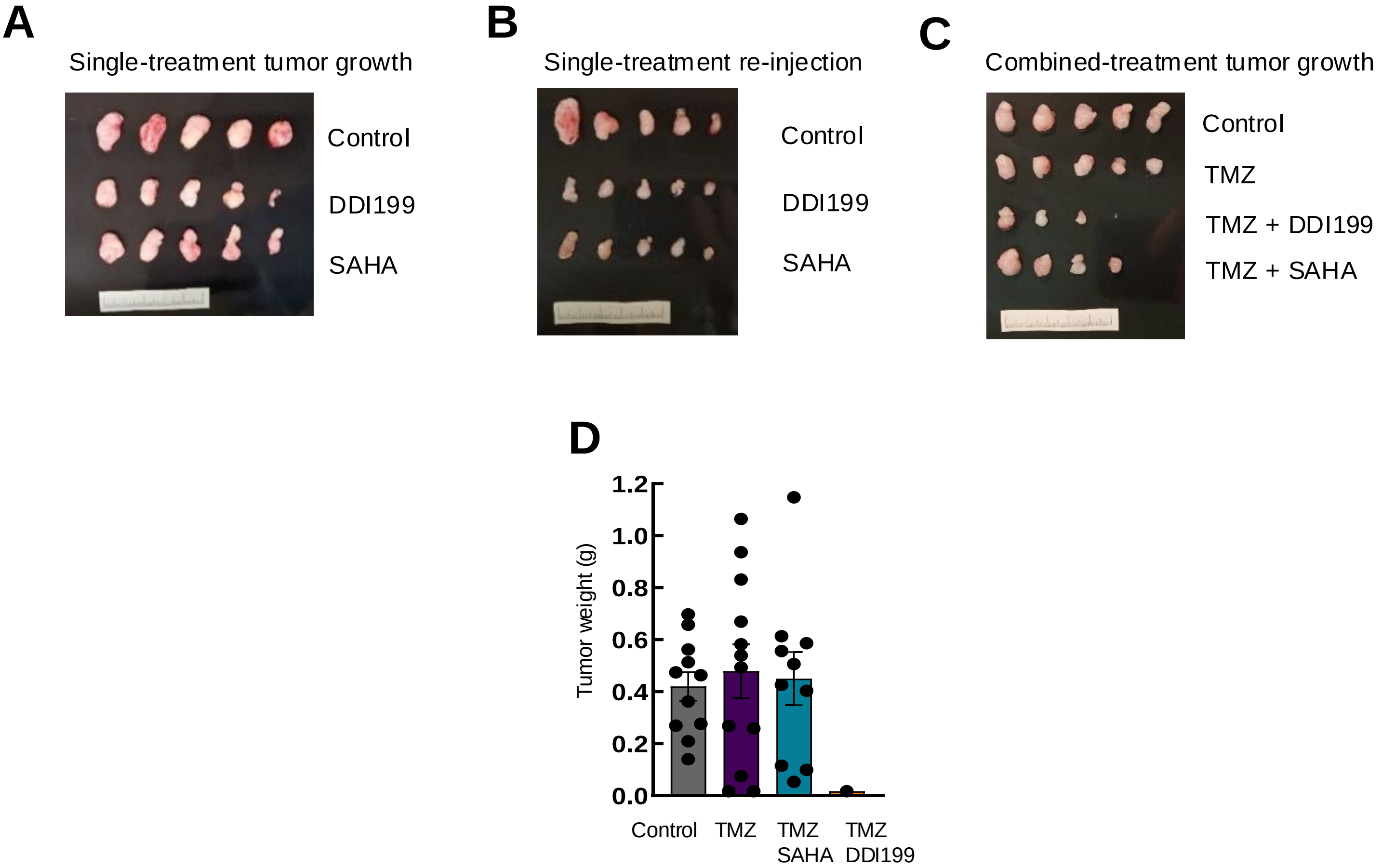

Suppl. Fig. 7 A) Representative images of subcutaneous tumors from single-treatment tumor growth experiment. **B)** Representative images of subcutaneous tumors from single-treatment re-injection experiment. **C)** Representative images of subcutaneous tumors from combined-treatment tumor growth experiment. **D)** Tumor weight at end-point day 36 of combined-treatment re-injection experiment.
