# Supplementary material for: Generation and validation of a novel multitarget small molecule in glioblastoma
Source: Cell Death Dis. 2025 Apr 4;16(1):250. doi: 10.1038/s41419-025-07569-1 (PMC11971462; doi:10.1038/s41419-025-07569-1)
Supplement: Supplementary file 3 — Original Data [file 41419_2025_7569_MOESM3_ESM.pptx]

## Slide 1
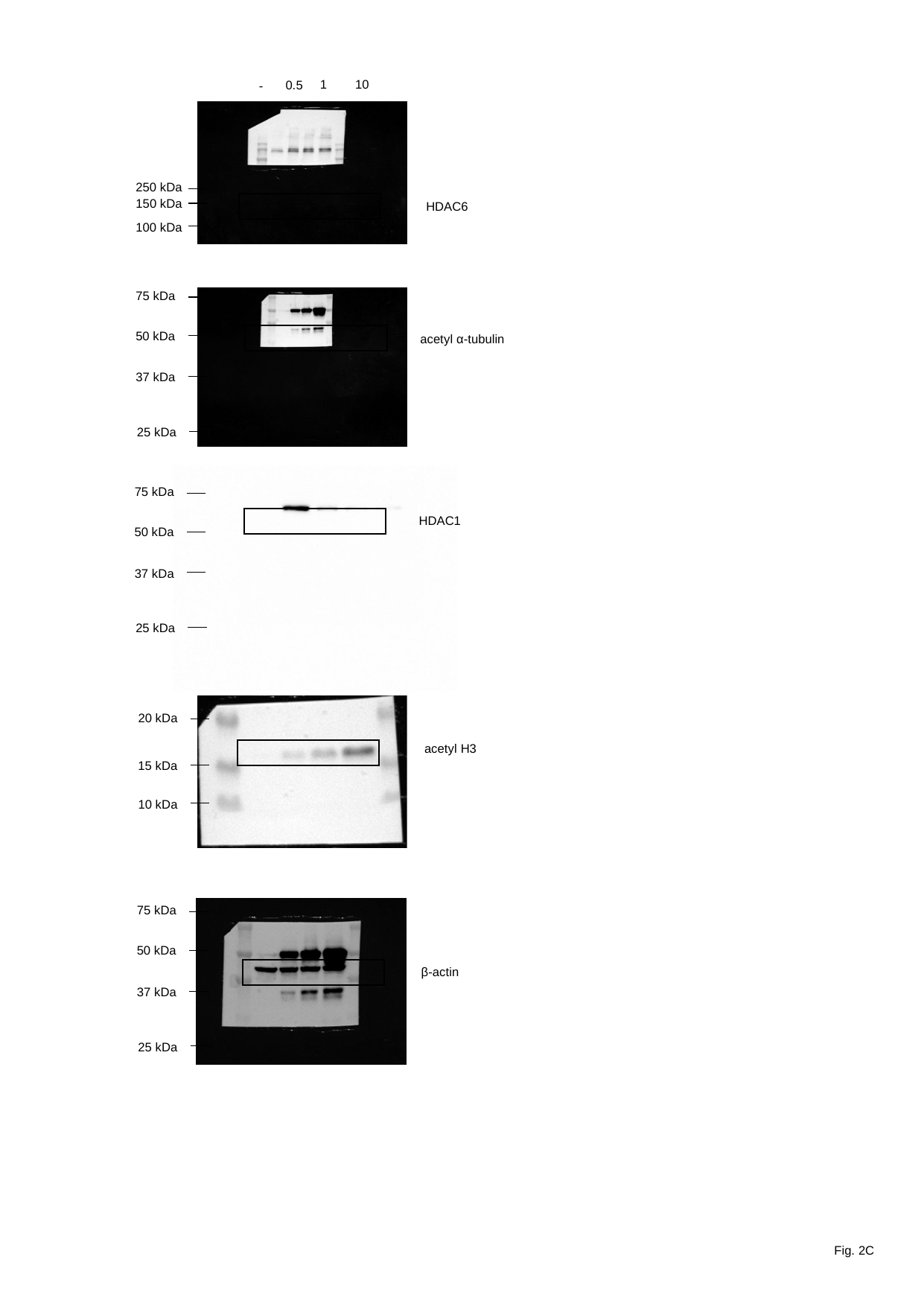

1
10
0.5
-
250 kDa
150 kDa
HDAC6
100 kDa
75 kDa
50 kDa
acetyl α-tubulin
37 kDa
25 kDa
75 kDa
HDAC1
50 kDa
37 kDa
25 kDa
20 kDa
acetyl H3
15 kDa
10 kDa
75 kDa
50 kDa
β-actin
37 kDa
25 kDa
Fig. 2C

## Slide 2
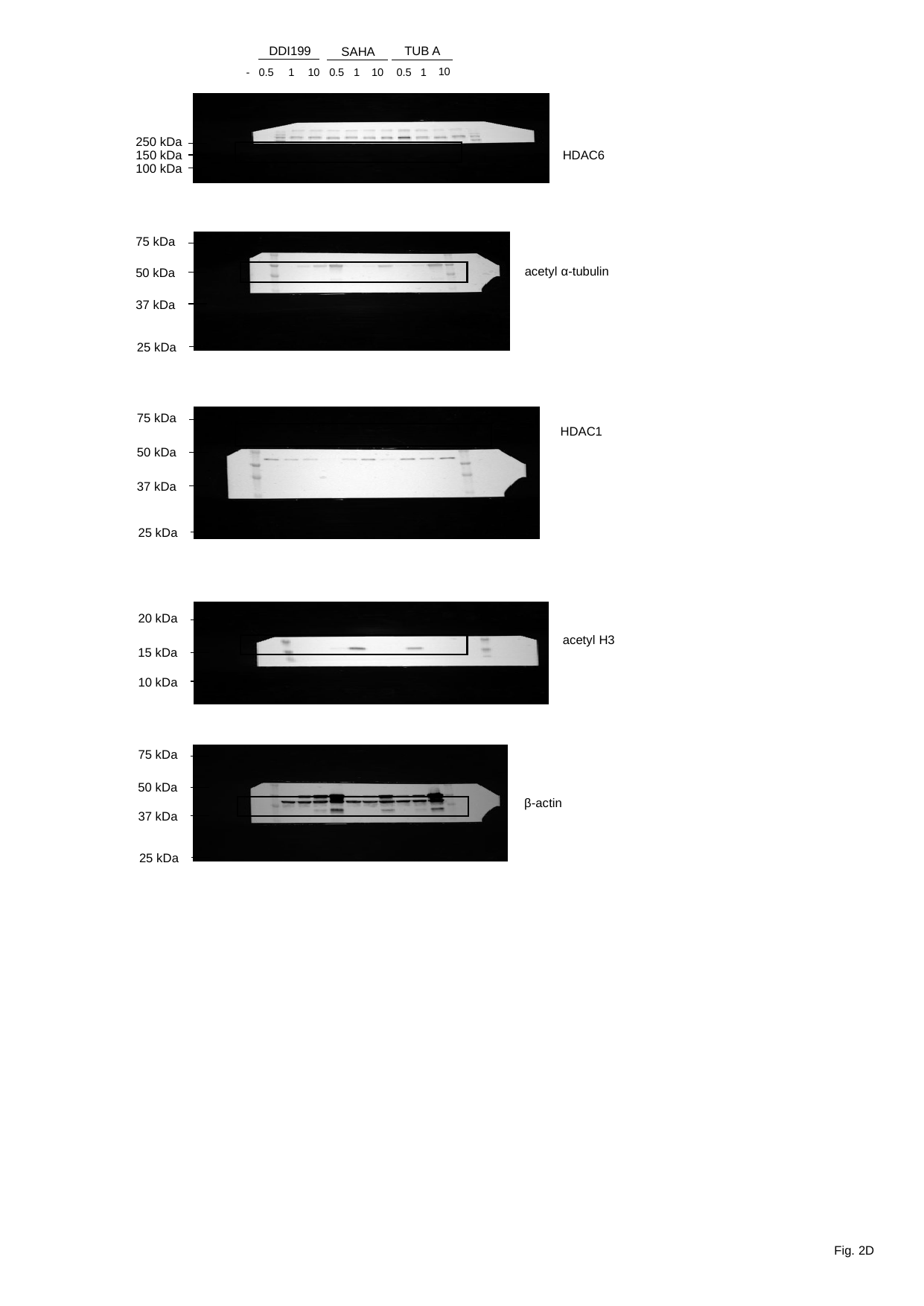

DDI199
TUB A
SAHA
10
0.5
1
0.5
1
-
0.5
1
10
10
250 kDa
150 kDa
HDAC6
100 kDa
75 kDa
acetyl α-tubulin
50 kDa
37 kDa
25 kDa
75 kDa
HDAC1
50 kDa
37 kDa
25 kDa
20 kDa
acetyl H3
15 kDa
10 kDa
75 kDa
50 kDa
β-actin
37 kDa
25 kDa
Fig. 2D

## Slide 3
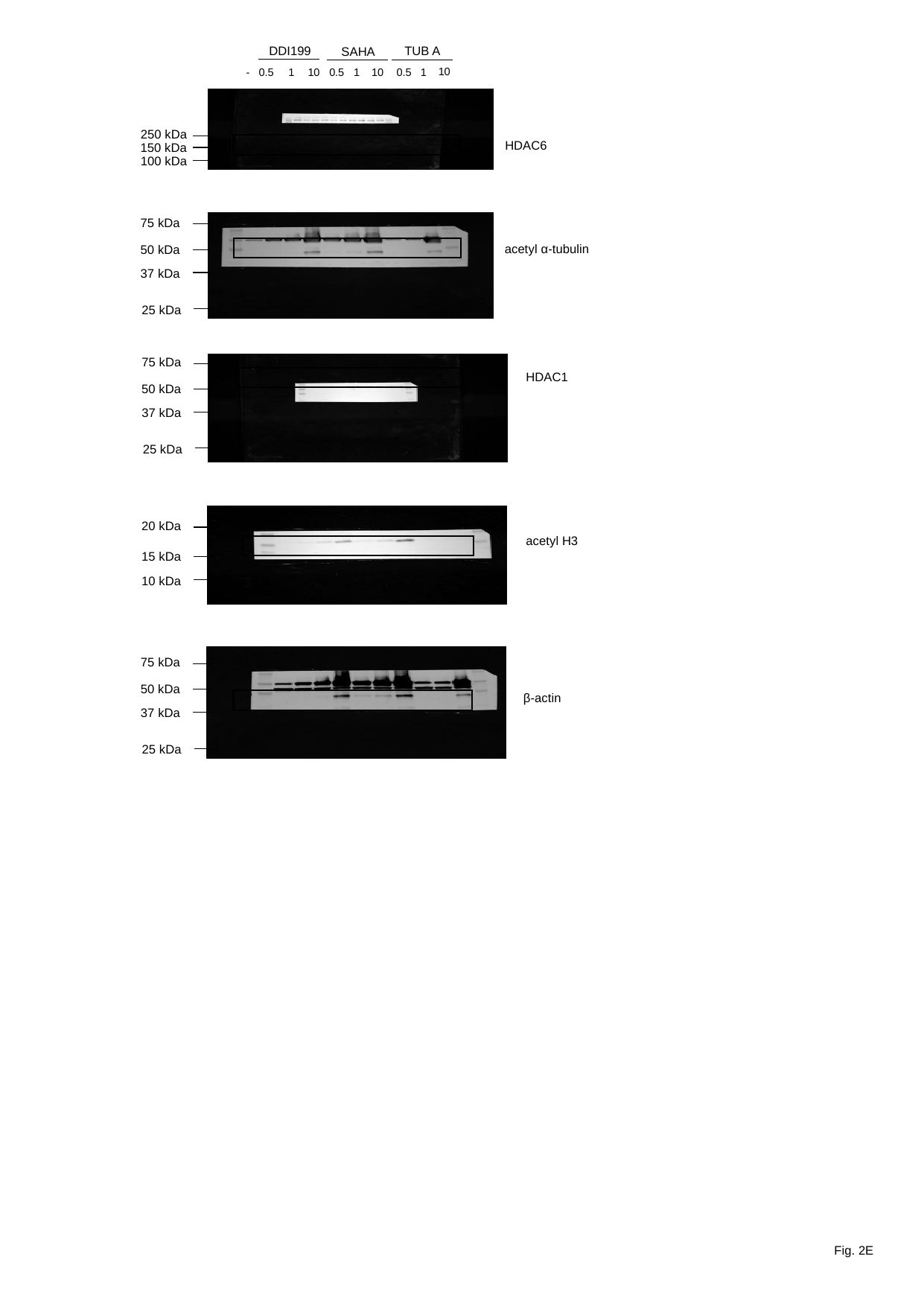

DDI199
TUB A
SAHA
10
0.5
1
0.5
1
-
0.5
1
10
10
250 kDa
HDAC6
150 kDa
100 kDa
75 kDa
acetyl α-tubulin
50 kDa
37 kDa
25 kDa
75 kDa
HDAC1
50 kDa
37 kDa
25 kDa
20 kDa
acetyl H3
15 kDa
10 kDa
75 kDa
50 kDa
β-actin
37 kDa
25 kDa
Fig. 2E

## Slide 4
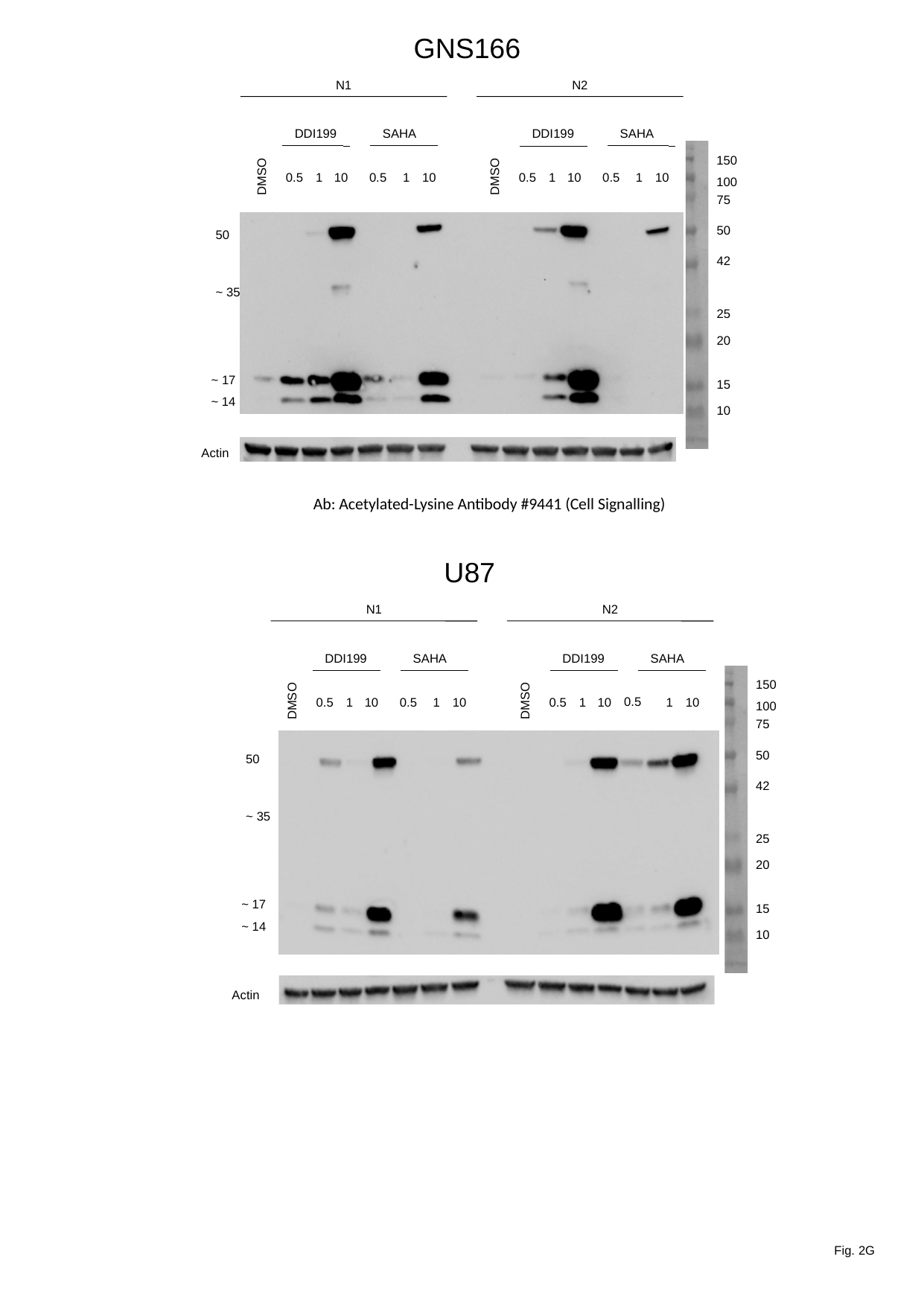

GNS166
N1
N2
SAHA
DDI199
SAHA
DDI199
150
DMSO
DMSO
0.5
0.5
0.5
1
1
1
10
0.5
1
10
10
10
100
75
50
50
42
~ 35
25
20
~ 17
15
~ 14
10
Actin
Ab: Acetylated-Lysine Antibody #9441 (Cell Signalling)
U87
N1
N2
SAHA
DDI199
SAHA
DDI199
150
DMSO
DMSO
0.5
0.5
0.5
1
1
1
10
0.5
1
10
10
10
100
75
50
50
42
~ 35
25
20
~ 17
15
~ 14
10
Actin
Fig. 2G
